# Isolation of a Novel Plant Growth‐Promoting *Dyella* sp. From a Danish Natural Soil

**DOI:** 10.1111/1758-2229.70186

**Published:** 2025-09-10

**Authors:** Laura Dethier, J. Rasmus P. Jespersen, Jemma Lloyd, Elena Pupi, Ruochen Li, Wanru Zhou, Fang Liu, Yang Bai, Barbara Ann Halkier, Deyang Xu

**Affiliations:** ^1^ DynaMo Center, Department of Plant and Environmental Sciences Faculty of Science, University of Copenhagen Frederiksberg Denmark; ^2^ Chinese Academy of Sciences Institute of Genetics and Developmental Biology Beijing China; ^3^ Peking‐Tsinghua Center for Life Sciences, College of Life Sciences Peking University Beijing China

**Keywords:** bacteria, microbe: higher organism interactions, microbiome

## Abstract

Natural soils are reservoirs of potentially beneficial microbes that can improve plant performance. Here, we isolated 75 bacterial strains from surface‐sterilised roots of *Arabidopsis thaliana* (Arabidopsis) grown in a natural soil derived from an alder swamp. Culture‐dependent isolation of individual strains from the roots, followed by monoassociation‐based screening, identified seven bacteria that promoted Arabidopsis seedling weight. Of those, we identified a new species from the *Dyella* genus which increased the biomass of Arabidopsis and tomato seedlings in agar, as well as the shoot biomass of Arabidopsis grown in both the alder swamp soil and potting soil. *Dyella* sp. A4 specifically promoted the elongation of lateral roots without affecting lateral root number and primary root elongation. The new *Dyella* sp. A4 expands the toolbox of biostimulants for plant growth promotion via modulating root architecture.

## Graphical abstract

Natural soils harbour untapped beneficial microbes. We screened for plant growth‐promoting bacteria isolated from *Arabidopsis* grown in an alder swamp soil collected in Danish nature and identified a new bacterial species, *Dyella* sp. A4, which modulates root architecture and enhances biomass of *Arabidopsis* and tomato.

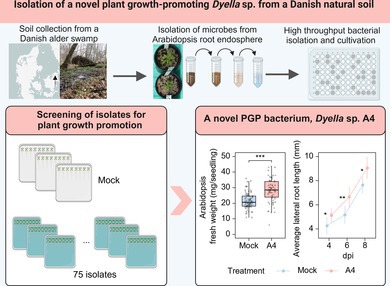

## 1 Introduction

Soils are inhabited by diverse microbial communities that vary in composition across different geographic locations, dependent on factors such as pH, nutrient availability, texture and moisture content (Fierer and Jackson 2006; Karimi et al. 2018; Xia et al. 2020). From the pool of microbes present in bulk soils, a subset is recruited to establish close associations with plant roots—collectively known as the root microbiota—resulting in a range of beneficial, neutral or pathogenic interactions with the host plant (Lundberg et al. 2012). The potential to harness beneficial members of the root microbiota to improve plant growth, nutrient acquisition and disease resistance has received considerable interest and investment in the last decade (Finkel et al. 2017; Zhang et al. 2019).

Natural soils represent a repository of beneficial microbes to be explored, particularly those from nutrient‐poor or arid soils, which often harbour plant growth‐promoting (PGP) taxa that can promote plant resilience to abiotic stresses (Eida et al. 2018; Khan et al. 2023). Examples include the bacterium *Isoptericola* sp. AK164 isolated from the rhizosphere of *Avicennia marina* plants grown on the Red Sea coast, which improves Arabidopsis biomass under both unstressed and salt‐stressed conditions (Alghamdi et al. 2023), and the root endophyte F229, abundant in a natural P‐limited soil, which promotes plant growth and shoot P accumulation in *Arabis alpina* (Almario et al. 2017). Soil nutrient status appears to influence not only which microbes are present, but also the nature of their interaction with the plant (Shi et al. 2021). Plants grown in nutrient‐rich soils are preferentially colonised by endophytes that produce phytohormones, while those in nutrient‐poor soils favour colonisation by nutrient‐solubilising bacteria in both the rhizosphere and endosphere (da Costa et al. 2014). For example, strains from the *Pandoraea* genus, which can solubilise P are more common in nutrient‐poor soils, in which they exhibited mostly endophytic behaviour (da Costa et al. 2014).

Root system architecture (RSA) is a key determinant of a plant's ability to explore and exploit soil nutrients, and its plasticity enables plants to optimise growth under heterogeneous or stressful environments (Giehl et al. 2014; Jones and Ljung 2012; Lavenus et al. 2013). A wide range of microbes have evolved to modulate root traits to the benefit of their host, enhancing nutrient absorption and accelerating plant growth (Khoso et al. 2024). Arbuscular mycorrhizal (AM) fungi, for example, not only solubilise and deliver phosphate via their extended hyphal networks (Smith et al. 2010), but also promote lateral root development, further enhancing colonisation and nutrient uptake (Chiu et al. 2022). Among bacteria, *Bacillus paralicheniformis* FMCH001 has been shown to promote root growth, nutrient uptake, and water use efficiency in soybean; inoculated plants showed comparable biomass while using ~23% less water under drought stress (Liu et al. 2023).

Despite many microbes promoting plant growth, few isolates have strong and reliable effects in soils. To address this, we explored the microbiota of a natural alder swamp soil which may harbour strains with robust PGP activity in soil. We first compared *Arabidopsis* performance in sterilised versus non‐sterilised soil to evaluate the contribution of the native microbiota to plant growth. We then combined culture‐independent profiling with a root‐derived culture collection and screened both individual isolates as well as a synthetic community for PGP activity. This led to the identification and characterisation of *Dyella* sp. A4, a novel bacterium that enhances biomass and specifically promotes lateral root elongation, without affecting primary root length and lateral root number.

## 2 Materials and Methods

### 2.1 Soil Collection and Processing

Soil was collected in March 2019 from the top 30 cm of an alder swamp located in a nature reserve in Denmark (55°58′05.6″ N, 12°16′16.7″ E). After air‐drying and sieving (10‐mm), half was distributed into hermetically sealed bags [300 mm × 600 mm × 0.08 mm (width × height × thickness)] and sterilised by gamma irradiation (18 kGy on each side; Sterigenics, Espergaerde, Denmark), and the rest remained non‐sterilised to retain the native microbiota. All soil was stored at 4°C and sterility was verified by plating soil suspensions onto nutrient agar media [0.5% peptone, 0.5% Bacto yeast extract, 0.5% NaCl, 1.5% g Bacto agar (BD Difco)] in triplicate. Soil physicochemical properties were analysed by Eurofins Agro (Denmark).

### 2.2 Plant Materials and Growth Conditions

Arabidopsis Col‐0 seeds were laboratory stocks, while DR5rev::GFP (CS9361) seeds were obtained from the Arabidopsis Biological Resource Center (ABRC). Seeds were surface‐sterilised (70% ethanol for 20 min, followed by water rinses) and stratified at 4°C in the dark before sowing. Arabidopsis plants were grown under long‐day conditions (16 h light, 140 μmol m^−2^ s^−1^, 22°C, 55%–60% relative humidity) in a growth chamber, unless stated otherwise. Tomato seeds ( *Solanum lycopersicum* cv. Moneymaker) were obtained from Frøsnapperen, surface‐sterilised (70% ethanol for 1 min, 0.5% NaOCl for 20 min, followed by water rinses) and grown under 16 h light, 100 μmol m^−2^ s^−1^, 25°C and 55% relative humidity in a growth chamber.

### 2.3 Growth of Arabidopsis in Sterilised and Non‐Sterilised Alder Swamp Soil

Arabidopsis seeds were sown into 9 cm pots filled with a 1:1 (vol/vol) mix of sterilised or non‐sterilised alder swamp soil and sand (0.71–1.25 mm). The two soil treatments were kept on separate tables to prevent cross‐contamination. Plants were grown in a greenhouse (16 h light/8 h dark) and the rosette area was quantified in ImageJ using colour thresholding.

### 2.4 Culture‐Independent Analysis of Root‐Associated Bacterial Communities From the Alder Swamp Soil

Arabidopsis was grown for 5 weeks in non‐sterilised alder swamp soil mixed with sand (1:1 vol/vol). Roots (endosphere and rhizosphere) were washed in phosphate buffered saline (PBS) and stored at −80°C along with unplanted soil aliquots. The experiment was conducted twice with seven roots and three soil replicates per experiment. DNA was isolated with the FastDNA Spin Kit for Soil (MP Biomedicals) and normalised to 3.5 ng μl^−1^ per sample. The V5–V7 region of the 16S rRNA gene was amplified in a two‐step PCR with primers 799F (5′‐AACMGGATTAGATACCCKG‐3′) and 1193R (5′‐ACGTCATCCCCACCTTCC‐3′) (Zhang et al. 2019), pooled, purified and sequenced on the Illumina HiSeq 2500 platform.

### 2.5 Isolation and Identification of Bacteria From Surface‐Sterilised Roots

Arabidopsis plants were grown for 8 weeks in non‐sterilised alder swamp soil mixed with sand (1:3 vol/vol). Roots were washed in deionised water, incubated in PBS (180 rpm, 15 min, 24°C) and surface‐sterilised with 0.25% NaOCl (30 s), followed by water rinses. Sterilisation was verified by plating the final rinse. Sterilised root tissue (20 mg) was homogenised in 10 mM MgCl_2_ and diluted into tryptic soy broth (TSB; Sigma‐Aldrich) in 96‐well plates (dilutions: 74×, 148×, 222×) using a limiting dilution approach (Zhang et al. 2021). After 2 weeks in the dark at 24°C, bacterial growth was observed in ~20% of wells at the 74× dilution. A total of 290 isolates were purified from the wells onto 0.5× TSB agar and identified via Sanger sequencing of ~1500 bp of the 16S rRNA gene using primers 27F (5′‐AGAGTTTGATCCTGGCTCAG‐3′) and 1492R (5′‐TACGGCTACCTTGTTACGACTT‐3′). Isolates with 100% sequence identity were considered redundant, and typically one representative was retained per group. However, in cases where isolates with identical sequences exhibited distinct colony morphologies, both were included to capture potential functional diversity. This resulted in a final culture collection of 75 unique isolates, which were preserved as 40% glycerol stocks at −80°C. Taxonomies were determined using the RDP 16S rRNA gene database. To infer phylogenetic relationships, sequences were aligned with MUSCLE 5.1 (Edgar 2004) and a maximum likelihood tree was constructed in PhyML 3.3.2 (Guindon et al. 2010) using a GTR model and 1000 bootstrap replicates. The consensus tree was visualised in iTOL (Letunic and Bork 2021).

### 2.6 Inoculation of Arabidopsis with the Full Culture Collection in Alder Swamp Soil

For the SynCom assay, isolates were inoculated in 600 μL of 0.5× TSB medium in a 96‐deep‐well plate and incubated with shaking (200 rpm, 28°C) for 5 days. Cultures were then diluted 1:10 in fresh 0.5× TSB and incubated for an additional 48 h. One strain (*Paracoccus* sp. H3) failed to grow and was excluded from this experiment. Cultures from both time points were pooled and normalised to equal OD_600_ values across the 74 members. The pooled mixture was pelleted (8000 g, 15 min), washed and resuspended to OD_600_ = 0.5 with sterile water. The bacterial suspension (100 μL) was diluted into 25 mL of deionised water and applied to 5.5 cm pots containing sterilised alder swamp soil mixed with sand (1:3 vol/vol). For the mock control, 100 μL of sterile water was added instead. Arabidopsis seeds were sown the following day and shoot biomass was recorded after 5 weeks.

### 2.7 Inoculation of Arabidopsis With Individual Isolates in Agar

For monoassociations, isolates were inoculated into agar following gnotobiotic growth protocols (Ma et al. 2022). Arabidopsis seeds were sown on 0.5× Murashige and Skoog (MS) agar (0.5× MS with vitamins (Duchefa), 0.5% sucrose, 1% Bacto agar, pH 5.7), stratified in the dark at 4°C for 2 days and germinated for 6 days. Individual bacterial strains were grown in 0.5× TSB medium (28°C, 200 rpm) for 1–14 days, washed and resuspended in 10 mM MgCl_2_. The bacterial suspensions were then added to warm (~50°C) 0.5× MS agar (1% Bacto agar, pH 5.7) just before it solidified, to a final OD_600_ of 0.0005. For axenic controls, 10 mM MgCl_2_ was added instead. The agar was poured into square plates and 6‐day‐old seedlings were transferred onto the solidified agar. Seedlings were grown for an additional 10 days before measuring total fresh weight.

### 2.8 PGP Assays With *Dyella* sp. A4

For soil assays, *Dyella* sp. A4 was suspended into 25 mL deionised water at OD_600_ concentrations of 0.0002, 0.0004 and 0.0008 (corresponding to ~3.3 × 10^5^, 6.6 × 10^5^ and 1.32 × 10^6^ CFU ml^−1^, respectively). Each suspension was applied to 5.5 cm pots containing sterilised alder swamp soil mixed with sand (1:3 vol/vol). Arabidopsis seeds were sown the next day and the shoot fresh weight was measured after 6 weeks. For a second experiment, A4 was prepared at an OD_600_ of 0.0002 and applied to non‐sterilised Pindstrup potting soil mixed with sand (1:3 vol/vol). Deionised water was used as the uninoculated control and shoot biomass was measured at 24 days post‐inoculation (dpi).

For RSA phenotyping, the strain was added to 0.5× MS agar (OD_600_ = 0.0005) before transferring Arabidopsis seedlings as described above. Primary root elongation was tracked by marking root tip positions after seedling transfer. Plates were imaged at 4, 6 and 8 dpi, and root traits were measured using EZ‐Rhizo software (Armengaud et al. 2009).

For monitoring auxin response, DR5rev::GFP seedlings were grown with A4 in the same way as WT Arabidopsis. Roots were stained with propidium iodide and imaged with a Leica SP5 X confocal microscope (60× objective) at 2 and 6 dpi. GFP and PI signals were detected at 500–530 nm and 570–630 nm, respectively.

For PGP assay with tomato, seeds were germinated on 0.5× MS agar (without sucrose) in the dark at 24°C for 3 days. The bacterium was added to 0.5× MS agar (OD_600_ = 0.000125) and poured into plant tissue culture boxes before transferring seedlings. Shoot fresh weight and shoot length were recorded at 24 dpi.

### 2.9 Root Colonisation Assay With Fluorescently‐Labelled *Dyella* sp. A4

A fluorescently labelled A4 strain (A4::Tn7‐mScarlet) was generated by chromosomal integration of mScarlet‐I using a mini‐Tn7 suicide plasmid (pMRE‐Tn7‐155) (Schlechter et al. 2018). To that end, the strain was grown to an OD_600_ of 0.4–0.6, washed and resuspended in 300 mM sucrose. Plasmid DNA (100 ng) was added to 50 μL of cells, followed by electroporation (2.5 kV cm^−1^, 25 μF, 200 Ω). After 2–3 h recovery in 0.5× TSB at 28°C, cells were plated on 0.5× TSB agar containing 50 μg ml^−1^ kanamycin. Colonies were re‐streaked without antibiotics and validated by PCR. Fluorescence was confirmed by mounting the strain in 1% agarose and imaging under a Leica DM5000B microscope at ×100 magnification. A4::Tn7‐mScarlet was inoculated in agar with Arabidopsis as previously described for the WT strain. Colonisation of the root was visualised using a Leica M205 FA stereo fluorescence microscope (ET mCherry filter; Leica Microsystems).

### 2.10 Whole‐Genome Sequencing of *Dyella* sp. A4 and Annotation

DNA was extracted from an overnight A4 culture using the FastDNA SPIN Kit for Soil (MP Biomedicals), followed by ethanol precipitation. Library preparation and sequencing were conducted by Novogene (Cambridge, UK) with the Illumina NovaSeq 6000 platform, generating ~9 million 150 bp paired‐end reads. Reads and adapters were filtered off using Trimmomatic (v.0.33) (Bolger et al. 2014) under SLIDINGWINDOW:4:20 MINLEN:50 and assembled using Spades (v.3.15.5) in isolate mode, resulting in 124 contigs. Genome quality was assessed using CheckM (v.1.2.1) and taxonomy was assigned using GTDB‐tk (v.2.0.0) based on R207 genome taxonomy database (GTDB). Species novelty was inferred by calculating pairwise distance between *Dyella* sp. A4 and the bacterial genome in R207 GTDB using Mash (2.3). The shortest Mash distance (0.076) exceeded 5%, supporting the classification of A4 as a novel species. To infer its functional potential, we predicted the coding sequence using prodigal (v.2.6.3) under single genome mode by setting −p to single. Functional characterisation was performed by blast against the KEGG database (2021 April version) using diamond v2.0.15 under the sensitive mode, and the e‐value cutoff was set to 1.0e−5. Only hits with identity larger than 50% were kept. Based on the KEGG gene and KO mapping information, the annotated genes were aggregated to KEGG orthologs (KO). To infer the strain's PGP potential, we curated a list of PGP traits based on published articles, including functions related to nutrient release, hormone biosynthesis and stress tolerance along with their KEGG ortholog information. These PGP traits were then summarised as present or absent within *Dyella* sp. A4.

### 2.11 In Vitro Characterisation of *Dyella* sp. A4 for PGP Traits

Inorganic phosphate solubilisation by *Dyella* sp. A4 was assessed by spot inoculation (OD_600_ = 0.1) on Pikovskaya's agar containing tricalcium phosphate, while nitrogen fixation was tested on Jensen's agar as described previously (Hazarika et al. 2021). Plates were incubated at 28°C for 7 days. Phosphate solubilisation index was calculated as total diameter (colony + halo)/colony diameter (Doilom et al. 2020). For IAA production, the strain was subcultured in 0.5× TSB medium supplemented with 0.15% L‐tryptophan for 1, 2, 4 and 6 days in the dark. Culture supernatants were mixed in a 1:1 ratio with Salkowski reagent (1 mL 0.5 M FeCl₃ in 49 mL 35% H₂SO₄) (Szkop et al. 2012) and incubated for 30 min in the dark before recording absorbance at 530 nm. IAA concentrations were calculated from a standard curve. Siderophore production was quantified using the Chrome Azurol S (CAS) assay as previously described (Alexander and Zuberer 1991; Payne 1994). The strain was grown in M9 minimal medium to stationary phase. Supernatant (500 μL) was mixed with 500 μL CAS solution and 10 μL shuttle solution, and absorbance was measured at 630 nm after a few minutes. Uninoculated M9 medium served as the blank. The reference (Ar) contained M9 medium, CAS reagent and shuttle solution, while the sample (As) contained bacterial supernatant instead of M9. Siderophore units were calculated as [(Ar—As)/Ar] × 100.

### 2.12 Statistical Analysis

Analyses were performed in R by a two‐sided *t*‐test, two‐tailed Mann–Whitney *U*‐test, or ANOVA followed by Tukey post hoc test to determine significance. A *p*‐value < 0.05 was taken as statistically significant.

## 3 Results

### 3.1 Native Microbiota in Alder Swamp Soil Contributes to Plant Growth

**FIGURE 1:**
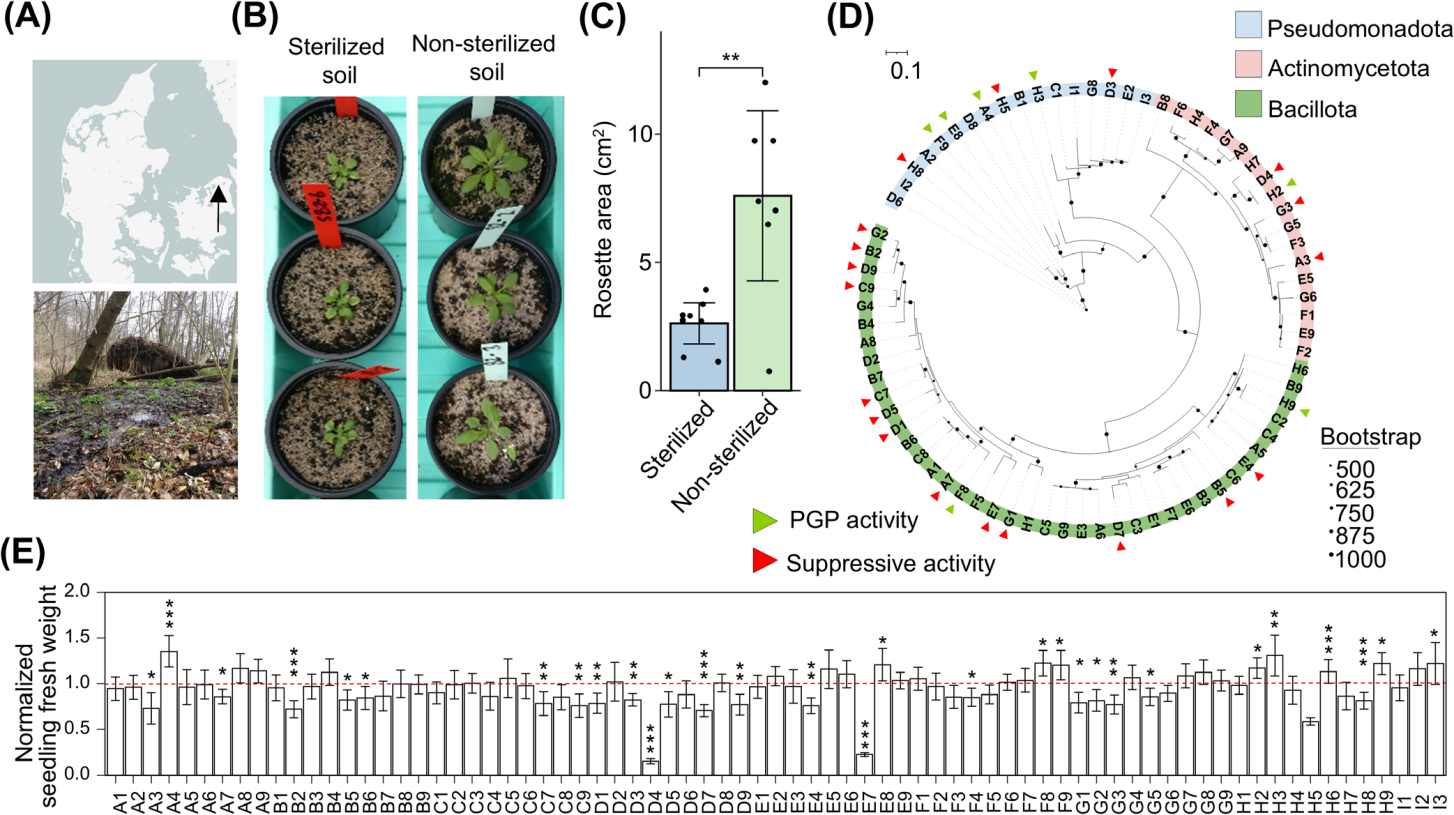
Microbiota isolated from a Danish alder swamp soil contribute to plant growth in agar. (A) Location (marked by a black arrow) and biotope of the alder swamp soil sampling site in Denmark. (B, C) Representative images (B) and rosette area (C) of Arabidopsis grown in sterilised and non‐sterilised alder swamp soil, respectively. (D) Consensus phylogenetic tree generated from the 16S rRNA gene sequences of 75 bacterial isolates. Tree tips are coloured according to phylum and black circles represent bootstrap values based on 1000 replicates. Green and red triangles depict strains with PGP and suppressive activity, respectively. (E) Fresh weight of Arabidopsis seedlings inoculated with the 75 strains in monoassociation normalised to axenic seedlings (indicated by a red dotted line). *n* = 15–24 seedlings per strain from one experiment. Data represent mean ±95% confidence interval in (E). Statistical significance was determined with a two‐sided *t*‐test in (C) and a two‐tailed Mann–Whitney U‐test in (E). *, ** and *** indicate *p* < 0.05, 0.01, 0.001, respectively.

We hypothesised that natural soils represent an untapped resource for discovering novel plant growth‐promoting (PGP) microbes. To test this, we collected organic soil from an alder swamp, which is dominated by alder ( *Alnus glutinosa* ), birch (*Betula* sp.) and sweet violet ( *Viola odorata* ) in Strødam Nature Reserve (North Zealand, Denmark) (Figure 1A). The soil is low in plant‐available macronutrients (Table S1), suggesting that it would be a promising source of beneficial microbes. To assess the role of the native microbiota in promoting growth, Arabidopsis was grown in either sterilised or non‐sterilised versions of the alder swamp soil. Soil sterilisation was performed using gamma irradiation, a method shown to effectively eliminate most fungal and bacterial groups while minimising disruption to soil physicochemical properties (Lees et al. 2018; McNamara et al. 2003). Consistent with this, total nitrogen, organic matter, macronutrients and pH remained largely unchanged after irradiation (Table S1). Soil sterility was confirmed by plating soil suspensions on nutrient agar, where no microbial growth was detected (Figure S1). Plants grown in non‐sterilised soil for 4 weeks developed significantly larger rosettes (7.6 ± 3.6 cm^2^) compared to those grown in sterilised soil (2.6 ± 1 cm^2^, *t*‐test, *p* < 0.01; Figure 1B,C), indicating that the native soil microbiota plays a key role in contributing to plant growth.

### 3.2 Culture‐Independent and Culture‐Dependent Profiling of Root‐Associated Bacterial Communities

To begin identifying the microbial drivers of the observed growth promotion, we profiled bacterial communities associated with Arabidopsis roots (endosphere and rhizoplane) and unplanted bulk soil after growing plants in non‐sterilised alder swamp soil. Unconstrained Principal Coordinate Analysis (PCoA) of Bray‐Curtis distances revealed a clustering of bacterial communities between root (i.e., endosphere and rhizoplane) and bulk soil (Figure S2A). Permutational multivariate analysis of variance (PERMANOVA) with Bray‐Curtis distances indicated that 66.64% of total variance could be explained by compartment (*p* < 0.01) (Table S2). Furthermore, bacterial alpha‐diversity based on the Shannon index was significantly higher in the bulk soil than in the root (Figure S2B). Root samples were predominantly composed of Pseudomonadota, Actinomycetota and Bacteroidota, with Pseudomonadota alone accounting for over half of the relative abundance (Figure S2C). Comparison of bacterial community composition at the phylum level revealed an enrichment of Bacteroidota, Pseudomonadota and Chloroflexota and depletion of Acidobacteriota, Bacillota, Nitrospirota and Planctomycetota in the roots (Table S3). At the amplicon sequence variant (ASV) level, 134 ASVs were enriched and 144 ASVs were depleted in root samples (relative abundance > 0.1% in at least one sample, edgeR, generalised linear model, *p* < 0.05, FDR < 0.2; Table S4).

Having established that Arabidopsis recruits a distinct bacterial assemblage from the alder swamp soil, we next aimed to identify strains within the root microbiota responsible for the observed PGP effect. Endophytes are key drivers of PGP (Almario et al. 2017). To enrich for endophytic bacteria, we generated a culture collection comprised of 75 unique bacterial isolates from surface‐sterilised roots of Arabidopsis (Figure S3) grown in the alder swamp soil (Figure 1D and Table S5). The majority belonged to the Bacillota phylum (53%), comprising species from 10 genera including *Bacillus*, *Neobacillus*, *Paenibacillus*, *Priestia* and *Sporosarcina*. The Actinomycetota phylum accounted for 25% of the culture collection, with 14 genera such as *Microbacterium*, *Arthrobacter*, *Knoellia* and *Rhodococcus*. The remaining isolates (22%) were classified under the Pseudomonadota phylum and spanned 11 genera, including *Mesorhizobium*, *Paraburkholderia*, *Variovorax*, *Dyella* and *Pseudomonas*. By sequence alignment of the V5–V7 region (Table S6), we found that 72 strains of the 75 share > 97% 16S rRNA gene sequence identity to ASVs from the culture‐independent analysis (Table S7), suggesting that our culture collection is representative of the microbiota derived from the alder swamp soil.

### 3.3 Screening of Isolates for Plant Growth Promotion

We next asked whether our culture collection could reproduce the plant growth‐promoting effect observed in the soil. We assembled a synthetic community (SynCom) of 74 isolates (one was excluded due to poor growth) and inoculated it into sterilised alder swamp soil. Contrary to expectations, SynCom‐treated plants showed significantly reduced shoot biomass after 5 weeks (66.5 ± 14.1 mg) compared to uninoculated controls (95.0 ± 22.0 mg, Mann–Whitney *U*‐test, *p* < 0.001; Figure S4). We therefore hypothesised that the beneficial effects of individual strains might be masked by antagonistic interactions within the complex community.

**FIGURE 2:**
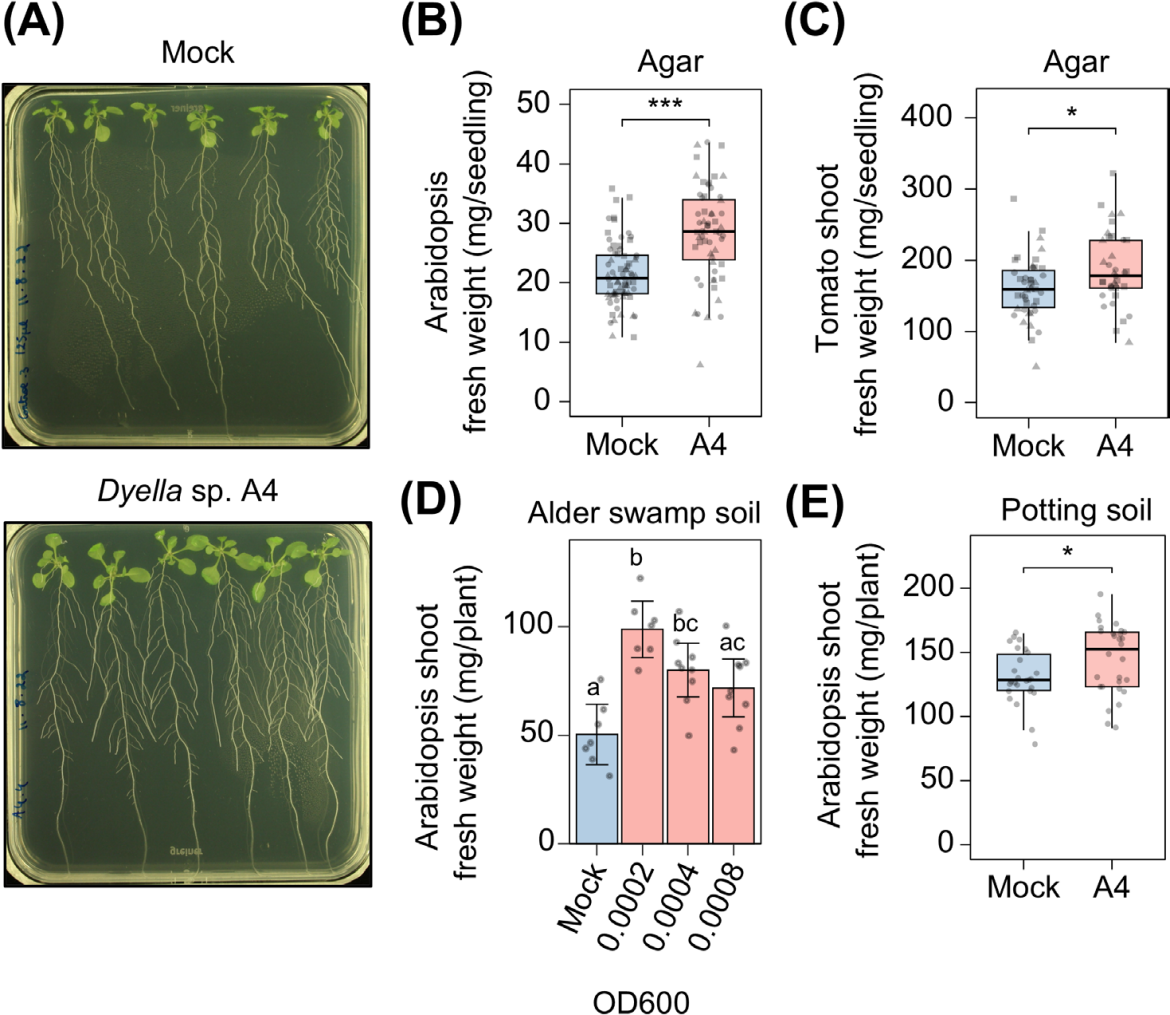
*Dyella* sp. A4 promotes growth of Arabidopsis and tomato. (A) Exemplary images of WT Arabidopsis growing in axenic agar (Mock) or agar inoculated with *Dyella* sp. A4. (B, C) Biomass of Arabidopsis (B) and shoots of tomato (C) seedlings grown on agar inoculated with *Dyella* sp. A4 compared to axenic plates (Mock). *n* = at least 59 plants per treatment in (B) and *n* = at least 37 plants per treatment in (C) pooled from three independent experiments (represented by different shapes). (D) Effect of *Dyella* sp. A4 on shoot fresh weight of Arabidopsis grown in sterilised alder swamp soil when inoculated at different concentrations (OD_600_). *n* = 7–9 plants per treatment. (E) Effect of *Dyella* sp. A4 at OD_600_ of 0.0002 on shoot biomass of Arabidopsis grown in potting soil. *n* = 26–28 plants per treatment. Data are presented as mean ±95% confidence interval in (D). Statistical significance was determined by two‐sided *t*‐test in (B, C and E) and by ANOVA followed by Tukey post hoc test in (D). * and *** indicate *p* < 0.05 and *p* < 0.001, respectively.

To disentangle individual contributions and identify candidate PGP bacteria, we subsequently screened all 75 isolates in monoassociation in agar plates, allowing for high‐throughput phenotyping. Six‐day‐old Arabidopsis seedlings were transferred to agar plates inoculated with each of the bacteria individually, and total biomass was measured at 10 days post‐inoculation (dpi). Seven of the 75 strains significantly enhanced seedling fresh weight compared to the axenic controls (Figure 1D,E). In contrast, 19 strains exhibited a suppressive effect on plant growth, with the majority (13 out of 19) belonging to the Bacillota phylum. The seven PGP strains were found across all three phyla represented in the culture collection: four belong to the Pseudomonadota (*Massilia* sp. F9; *Paraburkholderia* sp. E8; *Dyella* sp. A4; *Paracoccus* sp. H3), two to the Bacillota (*Paenibacillus* sp. F8; *Sporosarcina* sp. H9) and one to the Actinomycetota (*Arthrobacter* sp. H2). Among these, *Dyella* sp. A4 resulted in the highest increase in seedling biomass (27.6 ± 8.4 mg) relative to the mock (19.9 ± 5.6 mg, Mann–Whitney *U*‐test, *p* < 0.001) (Figure 1E). This growth promotion was reproducible across three independent experiments (Figure 2A,B). We next tested whether the strain's PGP potential could translate to another plant species. Inoculation of tomato seedlings with *Dyella* sp. A4 for 24 days in agar resulted in a higher shoot biomass (187.0 ± 51.5 mg) compared to the mock treatment (160.7 ± 42.4 mg, *t*‐test, *p* < 0.05; Figure 2C), indicating that the stimulation of plant growth is not limited to the Brassicaceae family.

### 3.4 *Dyella* sp. A4 Promotes Growth of Soil‐Grown Arabidopsis

To test whether *Dyella* sp. A4 also promotes growth in soil, Arabidopsis was grown in sterilised alder swamp soil inoculated with three different bacterial concentrations (OD_600_ = 0.0002, 0.0004 and 0.0008). Six weeks after inoculation, the lower and medium bacterial concentrations stimulated an increase in shoot biomass (98.7 ± 14.0 mg and 80.0 ± 16.1 mg, respectively) compared to the mock treatment (50.4 ± 15.0 mg, ANOVA, *p* < 0.05; Figure 2D). In contrast, the higher bacterial concentration showed no growth advantage, indicating a dose‐dependent effect consistent with previous studies (Jensen et al. 2024; Suckstorff and Berg 2003). Similarly, *Dyella* sp. A4 also promoted growth in potting soil, where the lower bacterial concentration (OD_600_ = 0.0002) significantly improved shoot fresh weight (144.7 ± 27.6 mg) relative to control plants (130.9 ± 21.1 mg, *t*‐test, *p* < 0.05; Figure 2E).

### 3.5 *Dyella* sp. A4 Promotes Lateral Root Elongation

**FIGURE 3:**
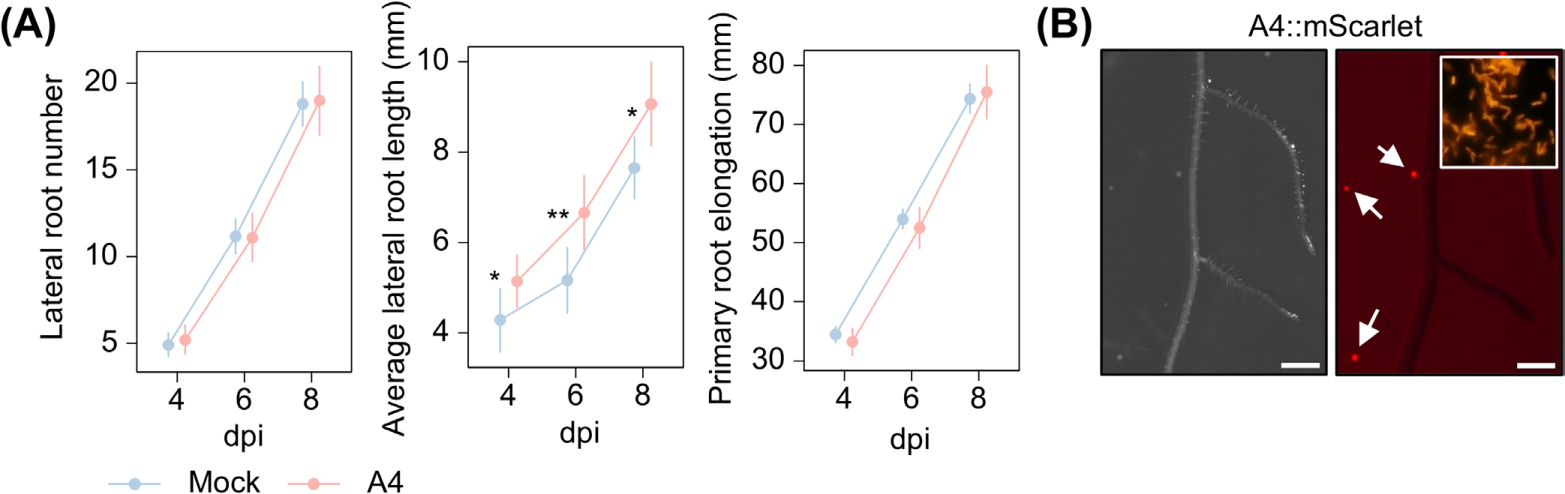
*Dyella* sp. A4 promotes lateral root elongation without colonising roots. (A) Effect of *Dyella* sp. A4 on root development at 4, 6 and 8 days post‐inoculation (dpi). *n* = at least 36 plants per treatment × time point combination pooled from two independent experiments. Data represent mean ±95% confidence interval. Statistical significance was determined with two‐tailed Mann–Whitney *U*‐test. * and ** indicate *p* < 0.05 and *p <* 0.01, respectively. (B) Stereo microscopy images of Arabidopsis roots grown on agar at 10 dpi with A4::mScarlet under brightfield view (left) and fluorescent signal from mCherry filter (right). Arrows indicate fluorescent A4::mScarlet colonies in agar without root association. Scale bar = 500 μm. Insert in the right panel shows fluorescence microscopy of A4::mScarlet cells from an overnight culture.

As the plasticity of the RSA is critical to maximise nutrient acquisition and plant growth, we monitored the root developmental response to *Dyella* sp. A4 at four, six and eight dpi. Inoculated seedlings showed longer average lateral root lengths (5.1 ± 1.7 mm at 4 dpi, 6.7 ± 2.4 mm at 6 dpi, 9.0 ± 2.7 mm at 8 dpi) compared to the mock (4.3 ± 2.4 mm at 4 dpi, 5.2 ± 2.5 mm at 6 dpi, 7.6 ± 2.3 mm at eight dpi), as determined by the Mann–Whitney *U*‐test (*p* < 0.05; Figure 3A). Interestingly, the number of lateral roots and primary root elongation were unaffected by the bacterial treatment (Figure 3A).

Auxin is a key regulator of RSA. To test whether the observed root phenotype is associated with changes in auxin response, we used the Arabidopsis DR5rev::GFP reporter line, in which DR5 activity reflects endogenous auxin levels in root tissues (Casimiro et al. 2001; Lewis et al. 2011). Fluorescence in the tip of primary roots was monitored after 2 and 6 days of inoculation. Consistent with the unchanged primary root elongation, *Dyella* sp. A4 did not alter DR5rev::GFP signal intensity at either time point compared to the mock treatment (Figure S5).

To investigate whether *Dyella* sp. A4 colonises Arabidopsis roots, we first constructed a fluorescently labelled version of the strain by integrating mScarlet into its genome (A4::mScarlet). Six‐day‐old Arabidopsis seedlings were then transferred onto agar inoculated with A4::mScarlet. The inoculated seedlings showed increased fresh weight (21.0 ± 5.3 mg) compared to the axenic controls (17.5 ± 4.4 mg, t‐test, *p* < 0.05; Figure S6), showing that A4::mScarlet kept its PGP activity. Although the strain expressed a strong fluorescent signal, no mScarlet signal was observed on the rhizoplane or the root interior (Figure 3B). We noticed, however, a few distinct fluorescent colonies in the surrounding agar medium (Figure 3B).

### 3.6 *Dyella* sp. A4 Is a New Plant Growth‐Promoting Species

To investigate the mechanisms behind *Dyella* sp. A4's PGP activity, we sequenced its whole genome, performed de novo assembly and predicted the coding sequences (CDSs). In total, 4373 CDSs were predicted and 4333 of those genes were complete genes. The predicted CDSs were annotated based on blast against the KEGG protein database. To annotate PGP‐related genes, we curated a collection of PGP‐related orthologs based on published literature, including genes involved in nitrogen fixation, phosphate solubilisation and mineralisation, siderophore biosynthesis and hormone modulation (auxin, cytokinin, gibberellins, salicylic acid and ACC deaminase). The strain was identified as a new species when compared against the currently available GTDB bacterial genome database and was found to harbour genes involved in organic phosphate mineralisation, for example, phosphodiesterase (K01126), phytase (K01093) and acid phosphatase (K09474), as well as hormone synthesis, for example, metK (K00789) (ethylene), miaA (K00791) and miaB (K06168) (cytokinin), tyrB (K00832) and ALHD (K00128) (auxin), although none of the complete biosynthetic pathways of hormones were present (Table S8).

To characterise the strain's capacity for nutrient solubilisation, nutrient mobilisation and hormone production, we assessed its ability to solubilise inorganic phosphate, fix nitrogen and produce the auxin indole‐3‐acetic acid (IAA) and siderophores in vitro. The strain solubilised inorganic phosphate as indicated by clear halos around colonies on Pikovskaya's agar medium (Figure S7A), with an average solubilisation index calculated as 1.32 ± 0.06 cm (Table S9). In contrast, it failed to grow on nitrogen‐free Jensen's medium (Figure S7B), suggesting an inability to fix nitrogen. The strain produced an average IAA concentration of 28.5 ± 2.23 μg mL^−1^ after 4 days of incubation with L‐tryptophan (Table S10) as detected by the Salkowski method, and secreted an average of 10.01% ± 1.87% siderophore units (Table S11) based on the CAS assay.

## 4 Discussion

In this report, we set out to identify beneficial bacteria from nature using soil from an alder swamp as a source. This led to the isolation of 75 bacterial strains, of which seven were growth‐promoting and 19 were growth‐suppressive in monoassociation. In parallel, culture‐independent profiling was conducted. We further characterised the bacteria *Dyella* sp. A4 exhibiting the highest PGP effect.

### 4.1 Root‐Associated Bacterial Communities of Arabidopsis Grown in a Natural Soil

We found that roots (endosphere and rhizoplane) of Arabidopsis grown in non‐sterilised alder swamp soil harbour distinct bacterial communities compared to bulk soil using a culture‐independent method (Figure S2). Previous studies have reported a decline in bacterial diversity from bulk soil to the rhizosphere and endosphere (Fitzpatrick et al. 2018; Ling et al. 2022; Mukhtar et al. 2021). In line with this pattern, we observed that the alpha‐diversity of root‐associated communities was lower than that of the bulk soil, suggesting a selective recruitment along the soil‐root continuum. This filtering is likely driven by root exudates which attract a specific subset of soil microbes to the rhizosphere, followed by host‐specific factors that shape the endosphere microbiota (Bulgarelli et al. 2013; Hardoim et al. 2015). We found roots to be dominated by members of Pseudomonadota, Actinomycetota and Bacteroidota, consistent with previous reports identifying these phyla as core taxa of the Arabidopsis root endosphere across various soil types (Bulgarelli et al. 2012; Lundberg et al. 2012). From surface‐sterilised roots, we retrieved 75 bacterial strains belonging to the phyla Bacillota (53%), Actinomycetota (25%) and Pseudomonadota (22%) (Figure 1D). This taxonomic distribution differs from a large‐scale study conducted by Levy et al. (2018), which cultured Arabidopsis root endophytes from plants grown in Mason Farm or Clayton soil/sand mixes. In that study, the majority of isolates belonged to Pseudomonadota (58%), followed by Actinomycetota (23%), Bacillota (15%) and a minority to Bacteroidota (4%) (Levy et al. 2018). These differences highlight the influence of soil origin in shaping root‐associated bacterial communities, although the same core phyla are present across distinct soil types.

### 4.2 *Dyella* sp. A4 Is a Novel Species Which Can Promote Plant Growth and Modulate Root Architecture

Arabidopsis grown in non‐sterilised alder swamp soil developed significantly larger rosettes than those in sterilised soil (Figure 1B,C), suggesting the presence of growth‐promoting microbes in the native soil microbiota. While inoculation of the individual strains altogether did not reproduce this growth‐promoting effect (Figure S4), monoassociation screening in agar identified seven strains with PGP activity (Figure 1E). Among these, *Dyella* sp. A4 showed the highest PGP effect and enhanced plant growth in both alder swamp and potting soils (Figure 2D,E).

Given that RSA is a key determinant of nutrient uptake and plant growth, we investigated how *Dyella* sp. A4 influences root development. In Arabidopsis, RSA is modulated by auxin, ethylene and cytokinin signalling pathways synergistically (Liu et al. 2017; Street et al. 2016). Many PGP bacteria have been found to modify RSA by modulating host hormone levels, either by producing hormones themselves or inducing hormone biosynthesis in the plant (Jensen et al. 2024; Xu et al. 2023). For instance, the rhizobacterium *Bacillus megaterium* strain WW1211 promotes lateral root initiation and shoot biomass by inducing auxin biosynthesis, redistribution and signalling in Arabidopsis (Wang et al. 2021).

Unlike many bacterial strains that promote lateral root formation while inhibiting primary root elongation in Arabidopsis (Spaepen et al. 2014; Wang et al. 2021; Zamioudis et al. 2013), *Dyella* sp. A4 specifically enhances lateral root elongation without altering primary root length or lateral root number (Figure 3A). The only similar RSA response we found so far is from *Bacillus subtilis* strain ALC_02, which produces the auxin IAA and induces an accumulation of auxin in both the shoot and root tissues of Arabidopsis (Jensen et al. 2024). IAA production has been reported for several previously isolated *Dyella* strains (Becerra‐Castro et al. 2011; Palaniappan et al. 2010). Although we did not identify the complete pathway for auxin biosynthesis in the genome of *Dyella* sp. A4, in vitro assays showed that the strain is capable of producing IAA in the presence of L‐tryptophan, with peak levels measured after 4 days (Table S10). However, no change in auxin response was observed in the Arabidopsis DR5rev::GFP reporter line at both 2 and 6 days post‐inoculation (Figure S5), suggesting that its effect on RSA does not involve major changes in auxin accumulation at the root tip.

Aside from auxin, certain bacteria promote plant growth by producing cytokinin or via aminocyclopropane‐1‐carboxylate (ACC) deaminase activity, which reduces plant ethylene levels (Noreen et al. 2012; Zerrouk et al. 2020). For example, the ACC deaminase‐producing strain *Variovorax paradoxus* 5C‐2 increases the leaf area and shoot biomass of *Arabidopsis* by acting on the plant's ethylene signalling pathway (Chen et al. 2013). Additionally, bacterial strains that modulate Arabidopsis root development through ethylene or cytokinin signalling instead of auxin have been reported (Gonin et al. 2023; López‐Bucio et al. 2007; Ortíz‐Castro et al. 2008). In our study, we did not identify the complete cytokinin biosynthetic pathway or ACC deaminase genes in the genome of *Dyella* sp. A4, making these mechanisms unlikely.

Interestingly, the strain promoted plant growth and lateral root elongation despite showing no evidence of root colonisation in plants grown on agar, suggesting that it may act through the emission of bioactive volatiles or the secretion of diffusible compounds into the medium. Several bacteria influence growth and RSA through cyclic peptides or volatile organic compounds (VOCs) (Dutta et al. 2025; Ortiz‐Castro et al. 2020; Zhang et al. 2007). For example, *Exiguobacterium* R2567 from the rice root microbiota produces the dipeptide cyclo(Leu‐Pro), which modulates plant architecture via strigolactone signalling (Zhang et al. 2025), while *Bacillus siamensis* YC7012 enhances Arabidopsis biomass and root number via VOCs independently of auxin, ethylene and jasmonic acid pathways (Hossain et al. 2019). Together, these findings point to several possible mechanisms for *Dyella* sp. A4's effect on RSA, which will be explored in future studies.

## Author Contributions

**Laura Dethier:** conceptualization, writing – original draft, writing – review and editing, visualization, data curation, investigation, project administration, methodology, validation, formal analysis. **J. Rasmus P. Jespersen:** investigation. **Jemma Lloyd:** investigation. **Elena Pupi:** investigation. **Ruochen Li:** investigation. **Wanru Zhou:** data curation, investigation. **Fang Liu:** investigation, data curation, writing – review and editing. **Yang Bai:** writing – review and editing, supervision, funding acquisition. **Barbara Ann Halkier:** supervision, writing – review and editing, conceptualization, funding acquisition. **Deyang Xu:** conceptualization, methodology, data curation, investigation, supervision, writing – original draft, writing – review and editing, visualization, project administration.

## Conflicts of Interest

The authors declare no conflicts of interest.

## Supporting information


**Figure S1:** Verification of efficiency of soil sterilisation.
**Figure S2:** Differentiation of soil and root microbiota from alder swamp soil.
**Figure S3:** Verification of efficiency of root‐surface sterilisation.
**Figure S4:** The synthetic community does not promote plant growth.
**Figure S5:** Monitoring the auxin response to *Dyella* sp. A4 in primary root tips.
**Figure S6:** A4::mScarlet promotes Arabidopsis growth.
**Figure S7:** Characterisation of phosphate solubilisation and nitrogen fixation by *Dyella* sp. A4.

**Table S1:** Physicochemical properties of γ‐irradiation‐sterilised and non‐sterilised alder swamp soil.
**Table S2:** PERMANOVA (Permutational Multivariate Analysis of Variance) using Bray–Curtis dissimilarity matrices.
**Table S3:** Different phylum between root and soil.
**Table S4:** Different ASVs between root and soil.
**Table S5:** Taxonomy of bacterial isolates.
**Table S6:** 16S rRNA gene sequences of bacterial isolates.
**Table S7:** 16S rRNA gene sequence similarity of cultivated bacteria and the corresponding ASVs.
**Table S8:** Annotation of PGP‐related orthologs in *Dyella* sp. A4 genome.
**Table S9:** Phosphate solubilisation by *Dyella* sp. A4.
**Table S10:** Quantification of IAA production by *Dyella* sp. A4.
**Table S11:** Quantification of siderophore production by *Dyella* sp. A4.

## Data Availability

The 16S rRNA sequences of bacterial isolates are provided in Table S6. The raw genome sequencing data of *Dyella* sp. A4 are openly available in the Zenodo database at DOI: 10.5281/zenodo.14673736.
