# Supplementary material for: Isolation of a Novel Plant Growth‐Promoting Dyella sp. From a Danish Natural Soil
Source: Environ Microbiol Rep. 2025 Sep 10;17(5):e70186. doi: 10.1111/1758-2229.70186 (PMC12421422; doi:10.1111/1758-2229.70186)
Supplement: Supplementary file 1 — Figure S1: Verification of efficiency of soil sterilisation. Figure S2: Differentiation of soil and root microbiota from alder swamp soil. Figure S3: Verification of efficiency of root‐surface sterilisation. Figure S4: The synthetic community does not promote plant growth. Figure S5: Monitoring the auxin response to Dyella sp. A4 in primary root tips. Figure S6: A4::mScarlet promotes Arabidopsis growth. Figure S7: Characterisation of phosphate solubilisation and nitrogen fixation by Dyella sp. A4. [file EMI4-17-e70186-s002.docx]

**Supporting information**

**Isolation of a novel plant growth-promoting *Dyella* sp. from a Danish natural soil**

Laura Dethier^1^, J. Rasmus P. Jespersen^1a^, Jemma Lloyd^1^, Elena Pupi^1b^, Wanru Zhou^2^, Fang Liu^2^, Yang Bai^3^, Barbara Ann Halkier^1^ & Deyang Xu^1*^

^1^DynaMo Center, Department of Plant and Environmental Sciences, Faculty of Science, University of Copenhagen, Frederiksberg, Denmark.

^2^ Institute of Genetics and Developmental Biology, Chinese Academy of Sciences, 100101 Beijing, China.

^3^ Peking-Tsinghua Center for Life Sciences, College of Life Sciences, Peking University, 100871 Beijing, China.

^a^Present address: NIRAS A/S, Sortemosevej 19, 3450 Allerød, Denmark.

^b^Present address: Fondazione Edmund Mach, Via E. Mach 1, 38098 San Michele All'Adige TN, Italy.

*Corresponding authors: [dyxu@plen.ku.dk](mailto:*dyxu@plen.ku.dk);

**Supporting figures**

**Figure S1.** Verification of efficiency of soil sterilization.

**Figure S2.** Differentiation of soil and root microbiota from alder swamp soil.

**Figure S3.** Verification of efficiency of root-surface sterilization.

**Figure S4.** The synthetic community does not promote plant growth.

**Figure S5.** Monitoring the auxin response to *Dyella* sp. A4 in primary root tips.

**Figure S6.** A4::mScarlet promotes Arabidopsis growth.

**Figure S7.** Characterization of phosphate solubilization and nitrogen fixation by *Dyella* sp. A4.


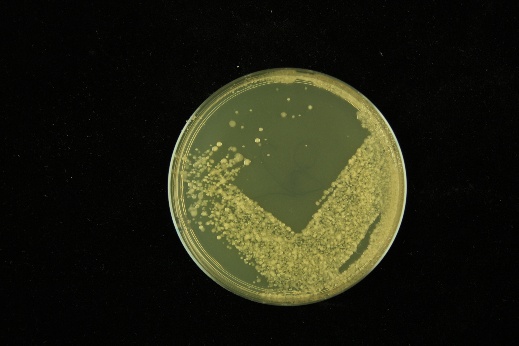

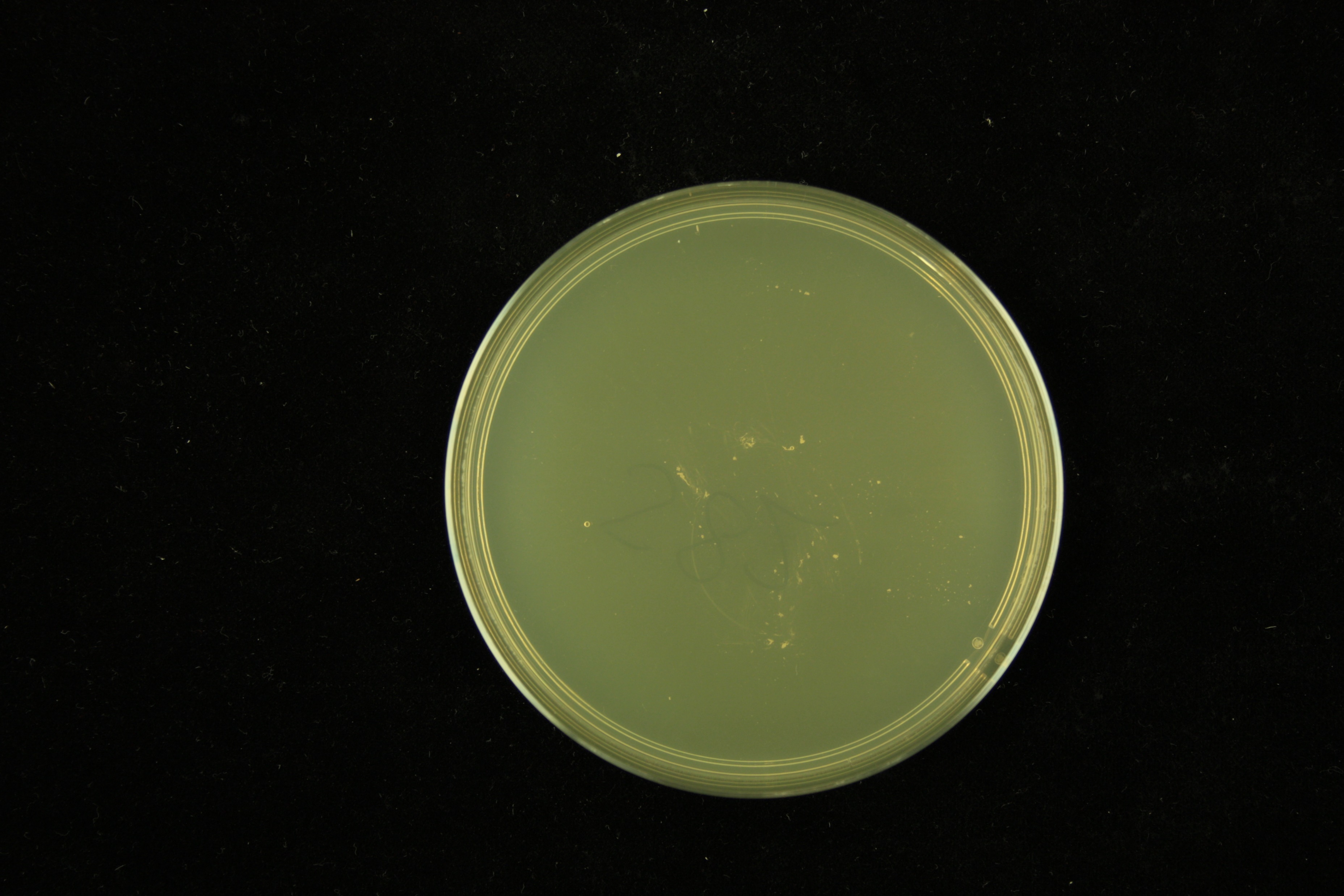

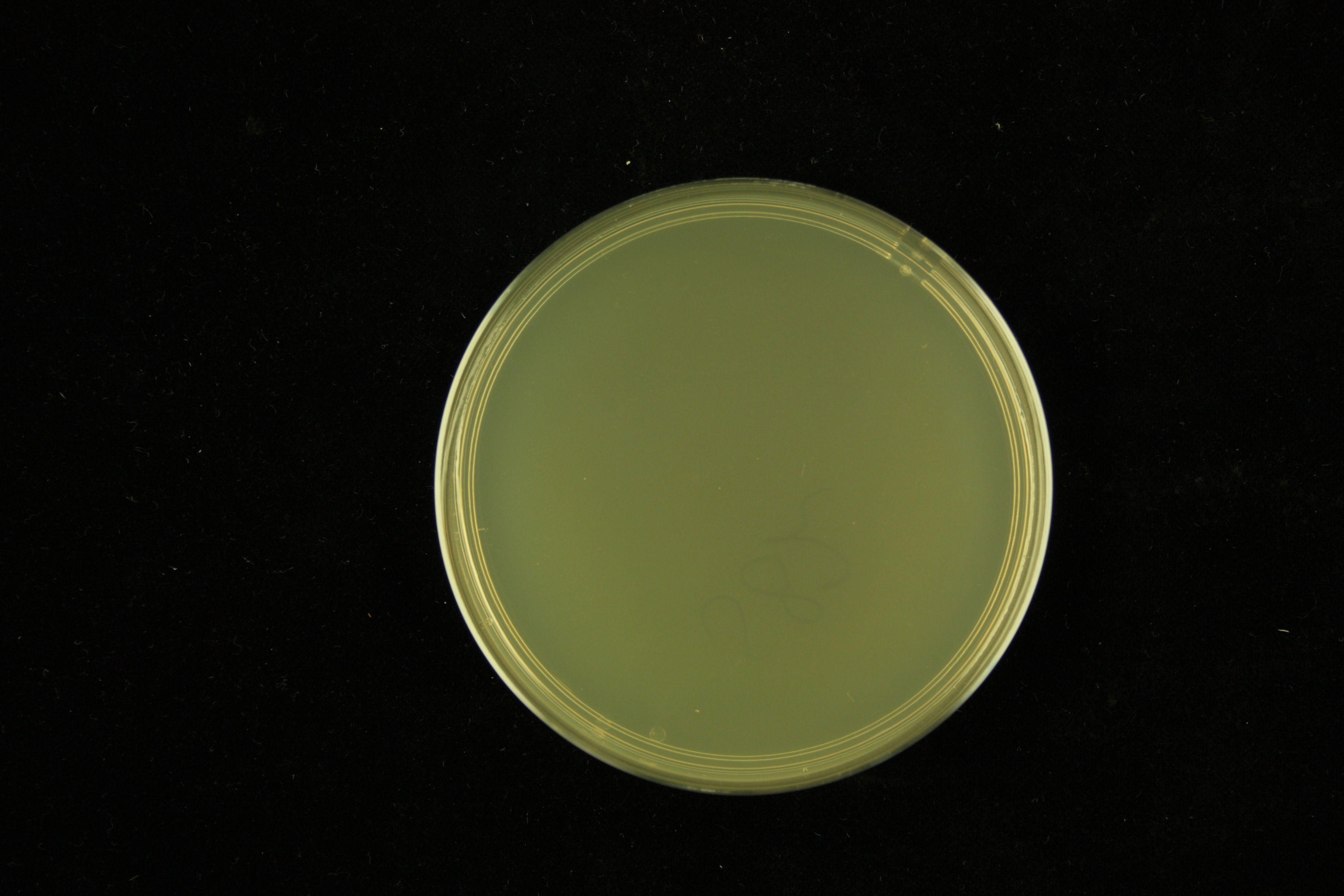

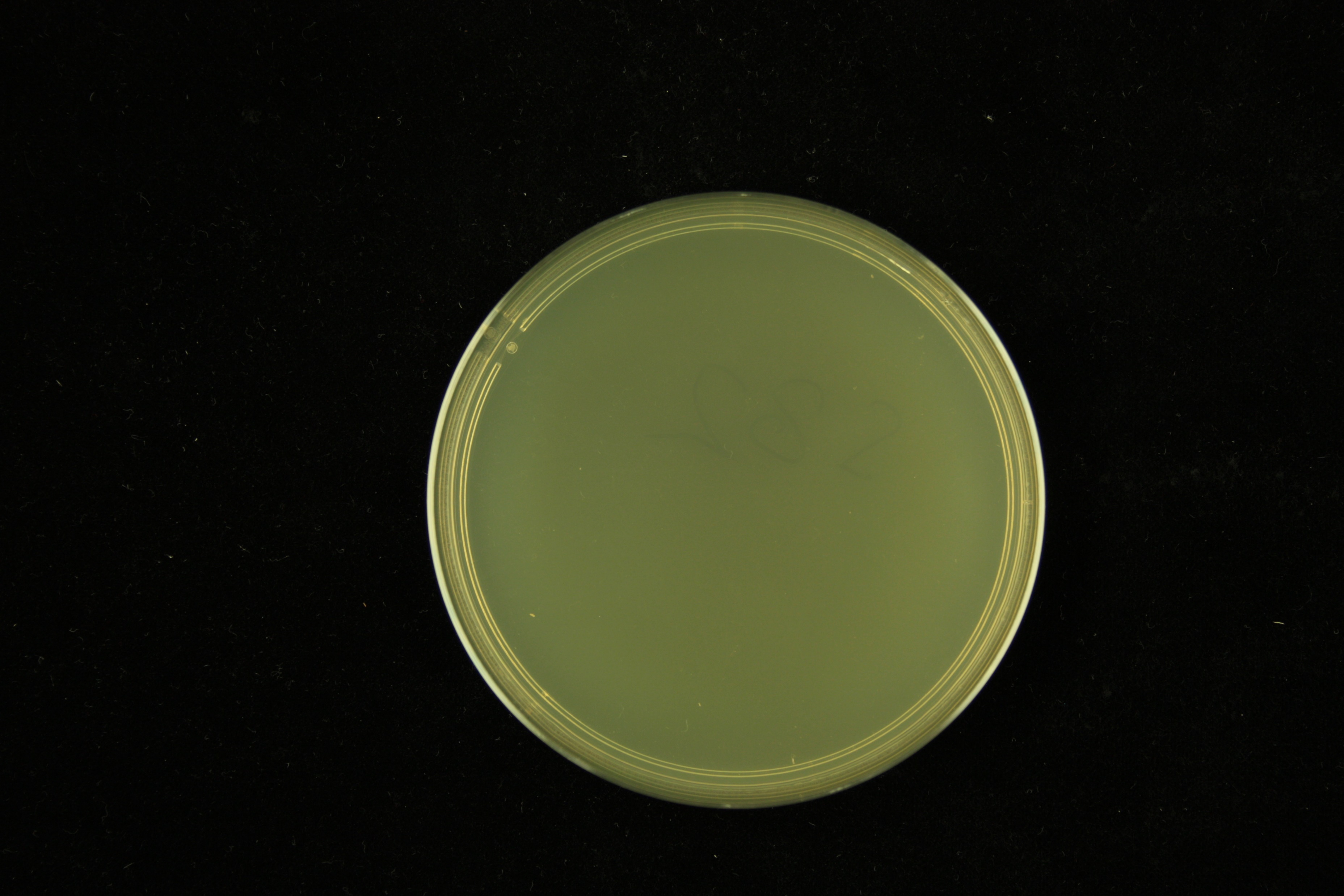

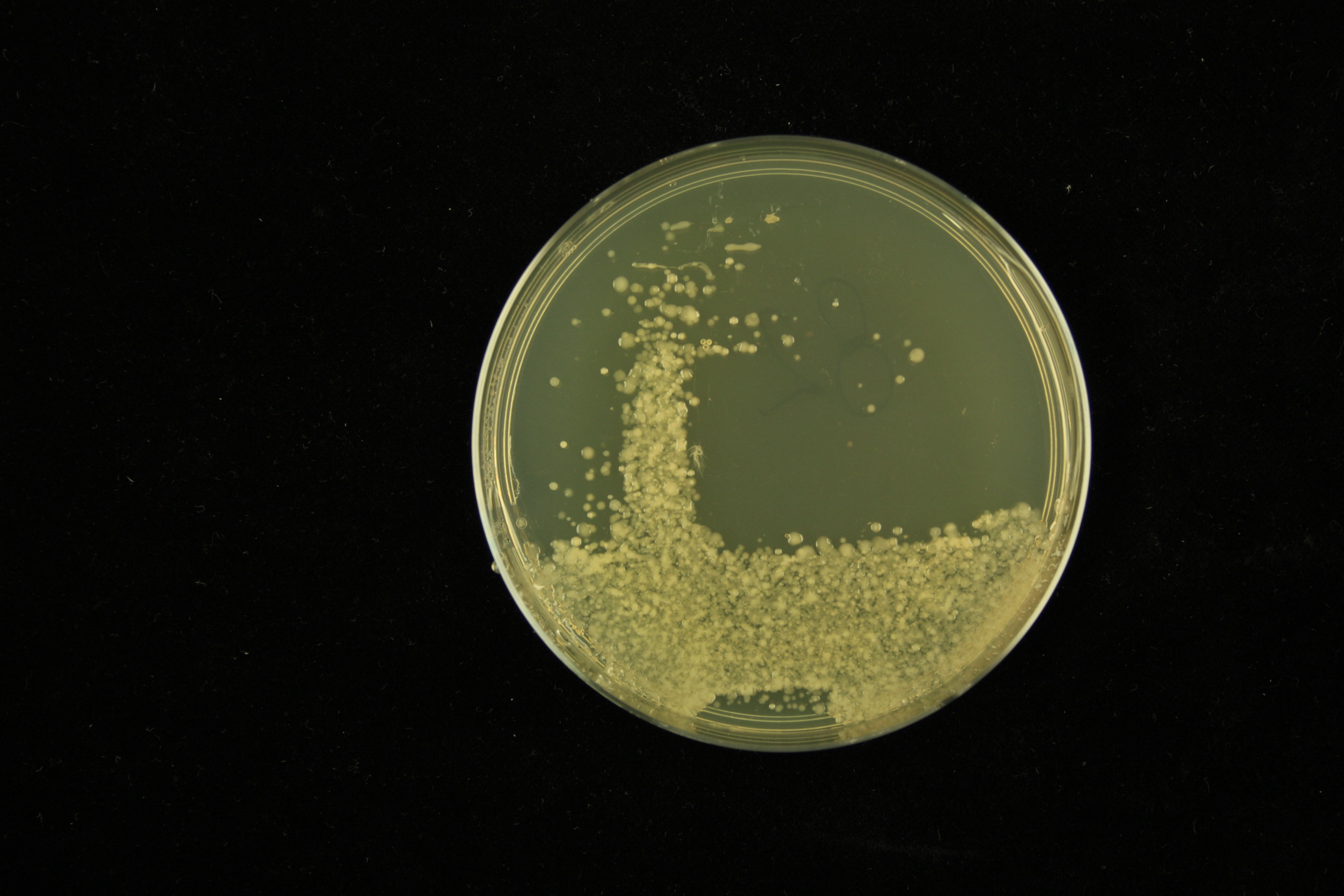

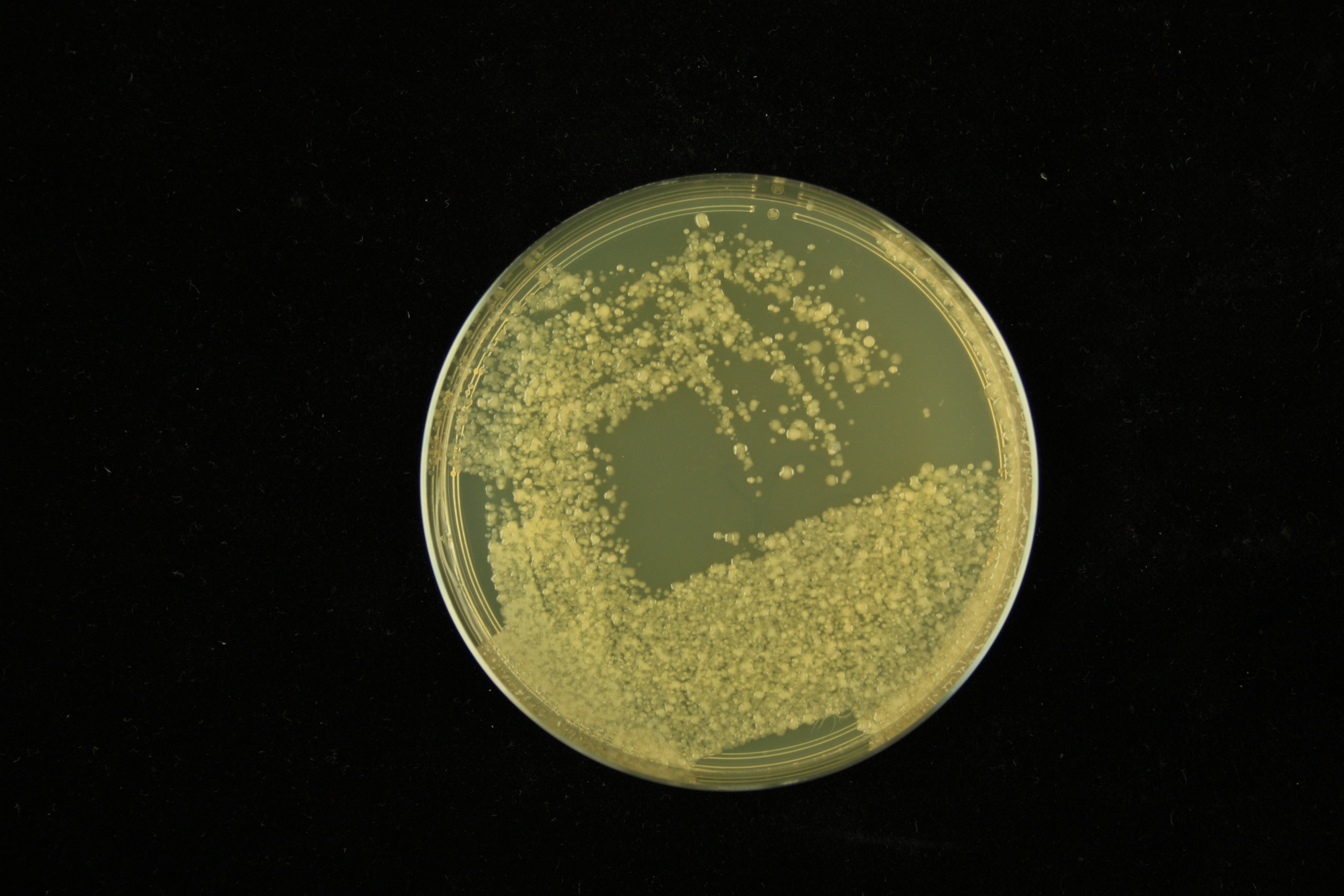


**Figure S1.** Verification of efficiency of soil sterilization. Nutrient agar plates streaked with γ-irradiation-sterilized alder swamp soil (top) and non-sterilized alder swamp soil (bottom) in triplicates.


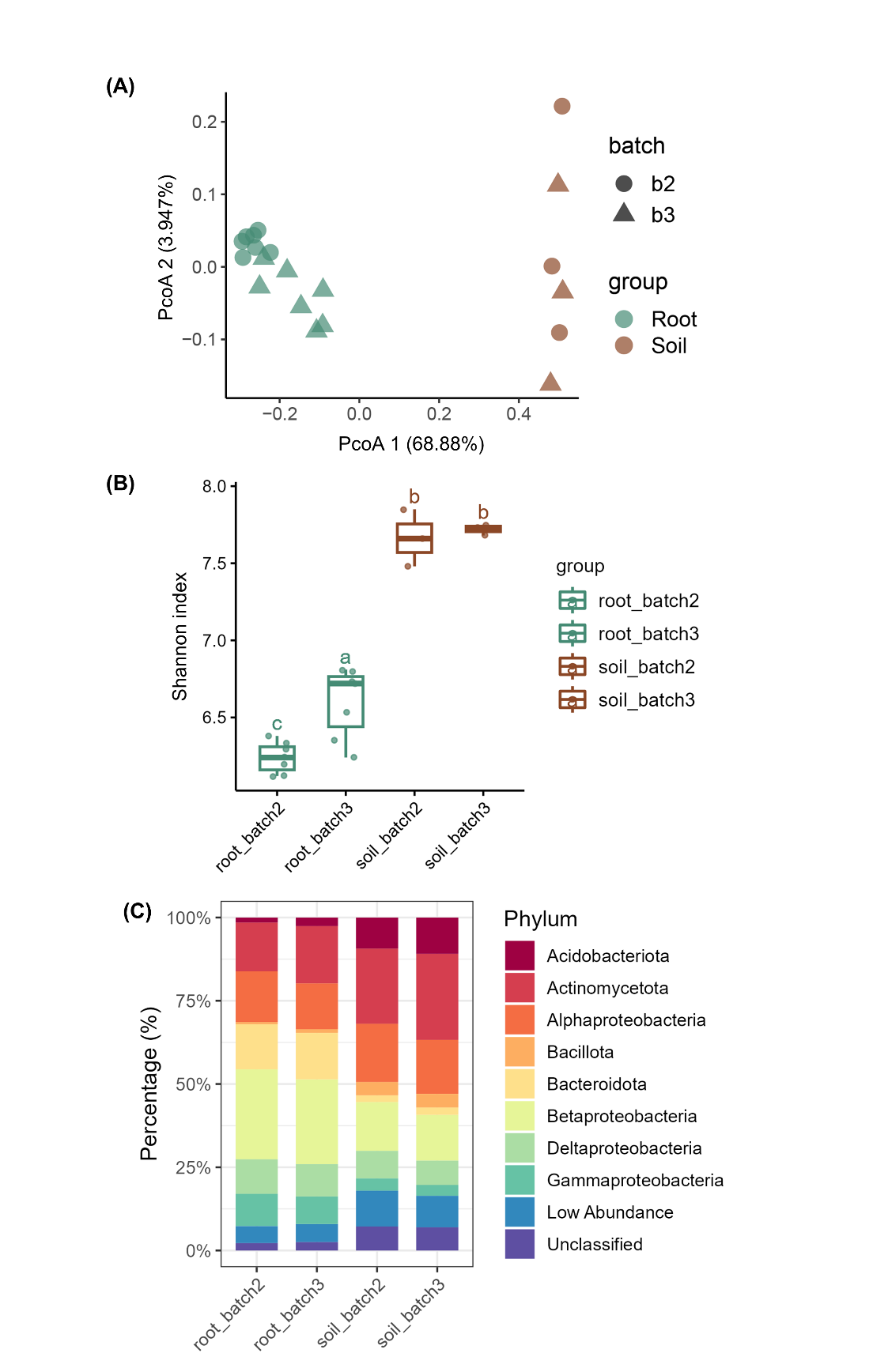


**Figure S2.** Differentiation of soil and root microbiota from alder swamp soil. (A, B) Principal Coordinate Analysis (PCoA) based on Bray-Curtis distances (A) and alpha-diversity based on Shannon index (B) for bacterial communities in root and soil samples. Different letters in (B) denote significant differences between groups (Tukey HSD test, FDR adjusted P < 0.05). (C) Relative abundance of bacterial phyla in soil and root samples. For each batch, n = 7 (roots) and n = 3 (soil).


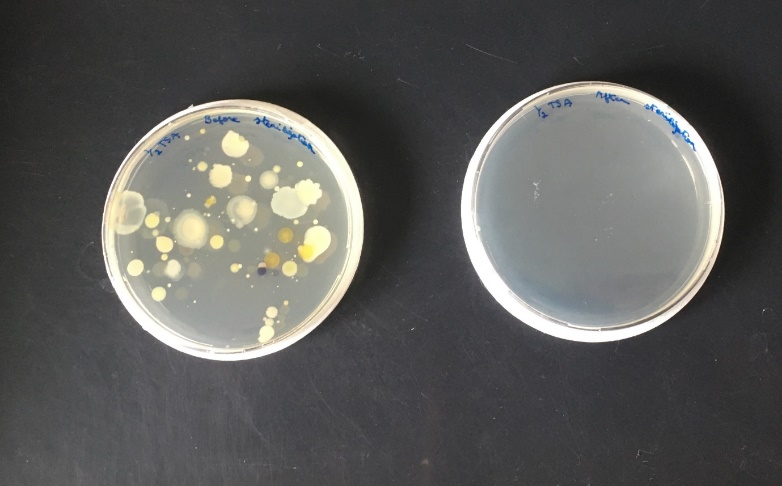

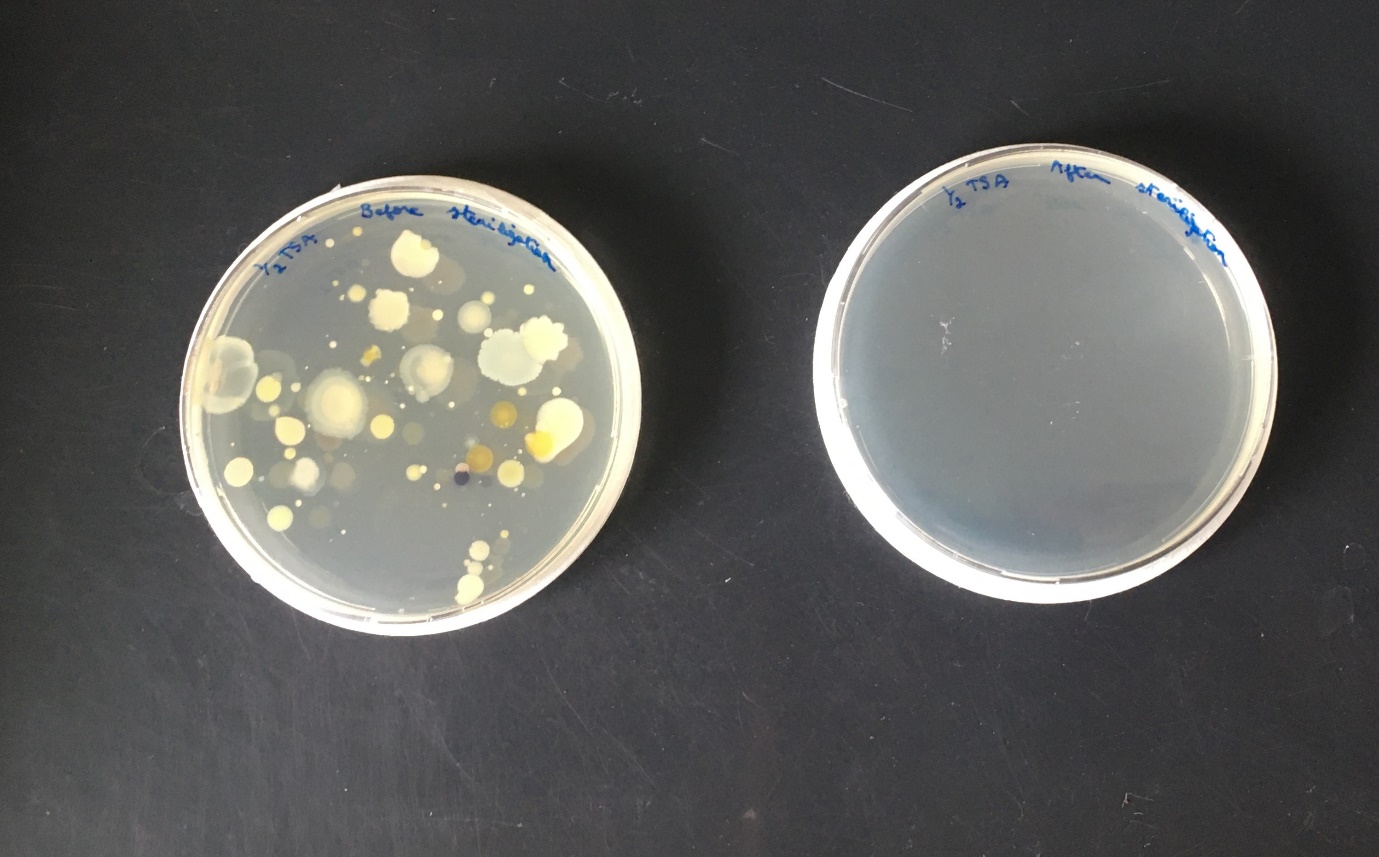


**Figure S3.** Verification of efficiency of root-surface sterilization. TSA plates streaked with water rinse from Arabidopsis roots before (left) and after (right) surface-sterilization.


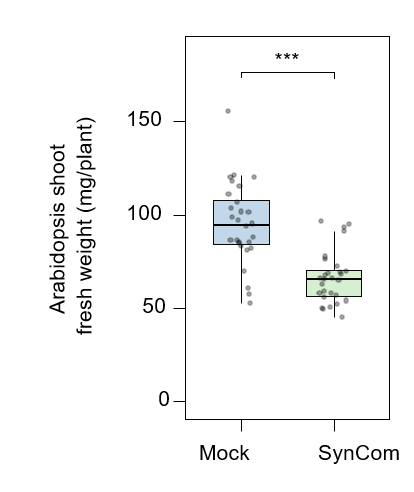


**Figure S4.** The synthetic community does not promote plant growth. Shoot biomass of Arabidopsis grown for five weeks in sterilized alder swamp inoculated with the 74-member SynCom compared to uninoculated controls (Mock). n = 28 plants per treatment from one experiment. Statistical significance was assessed using the Mann-Whitney U-test (*** indicates *P* < 0.001).


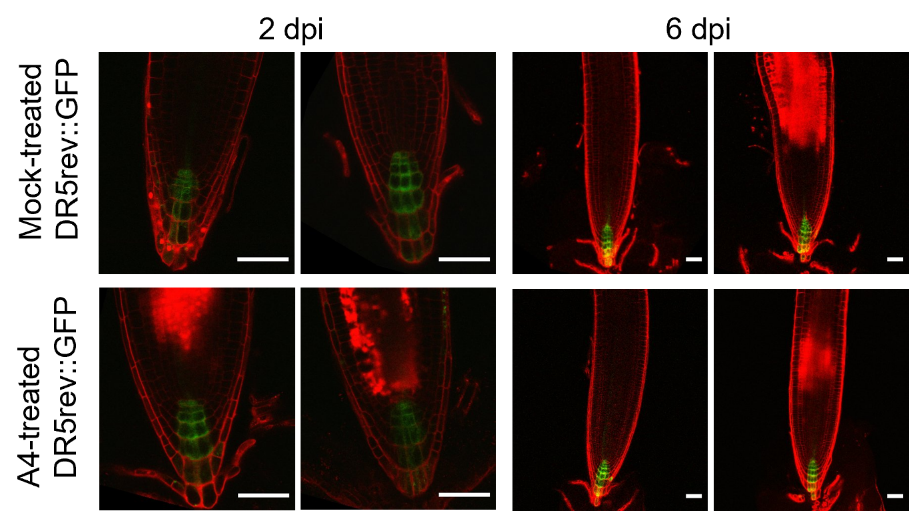


**Figure S5.** Monitoring the auxin response to *Dyella* sp. A4 in primary root tips. Confocal microscopy images of root tips from Arabidopsis DR5rev::GFP auxin reporter grown on axenic agar (top) or agar inoculated with *Dyella* sp. A4 (bottom) at two and six dpi. Two representative roots are shown per treatment and timepoint. Scale bars = 50 μm.


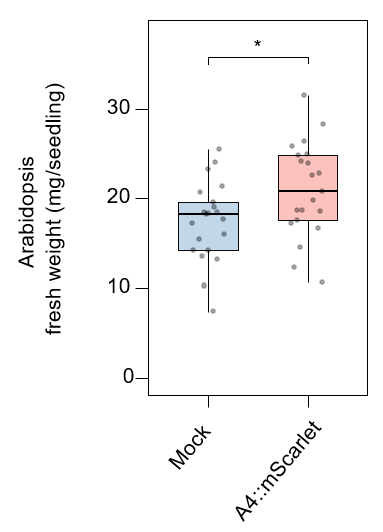


**Figure S6.** A4::mScarlet promotes Arabidopsis growth. Fresh weight of Arabidopsis seedlings grown on agar plates inoculated with A4::mScarlet for 10 days compared to axenic controls (Mock). n = 21 plants per treatment from one experiment. Significance was assessed via two-sided t-test (*indicates *P* < 0.05).

**(A)**

**(B)**


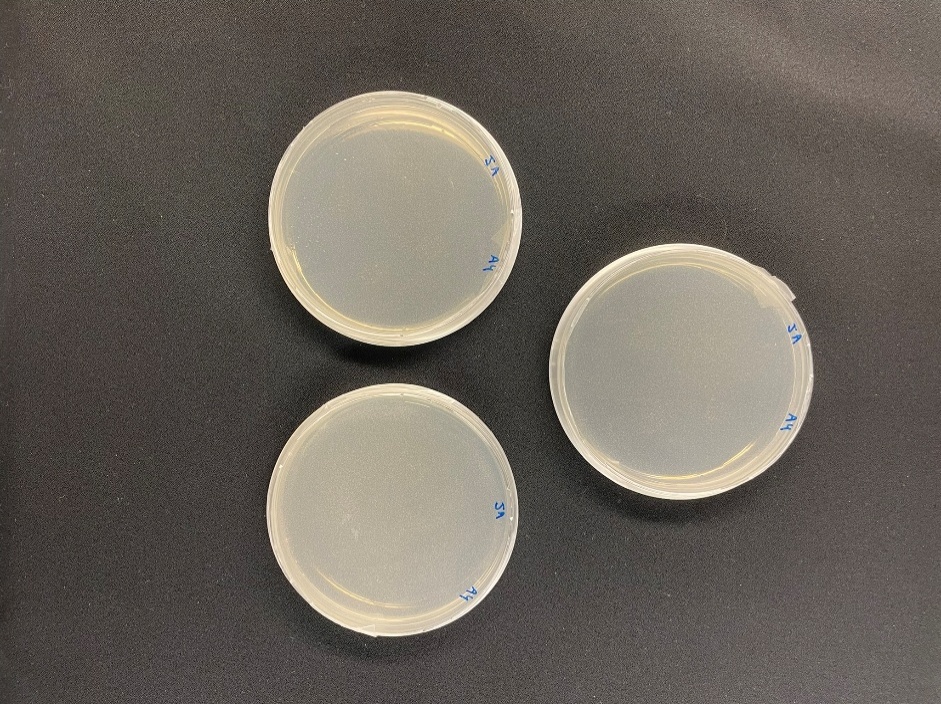

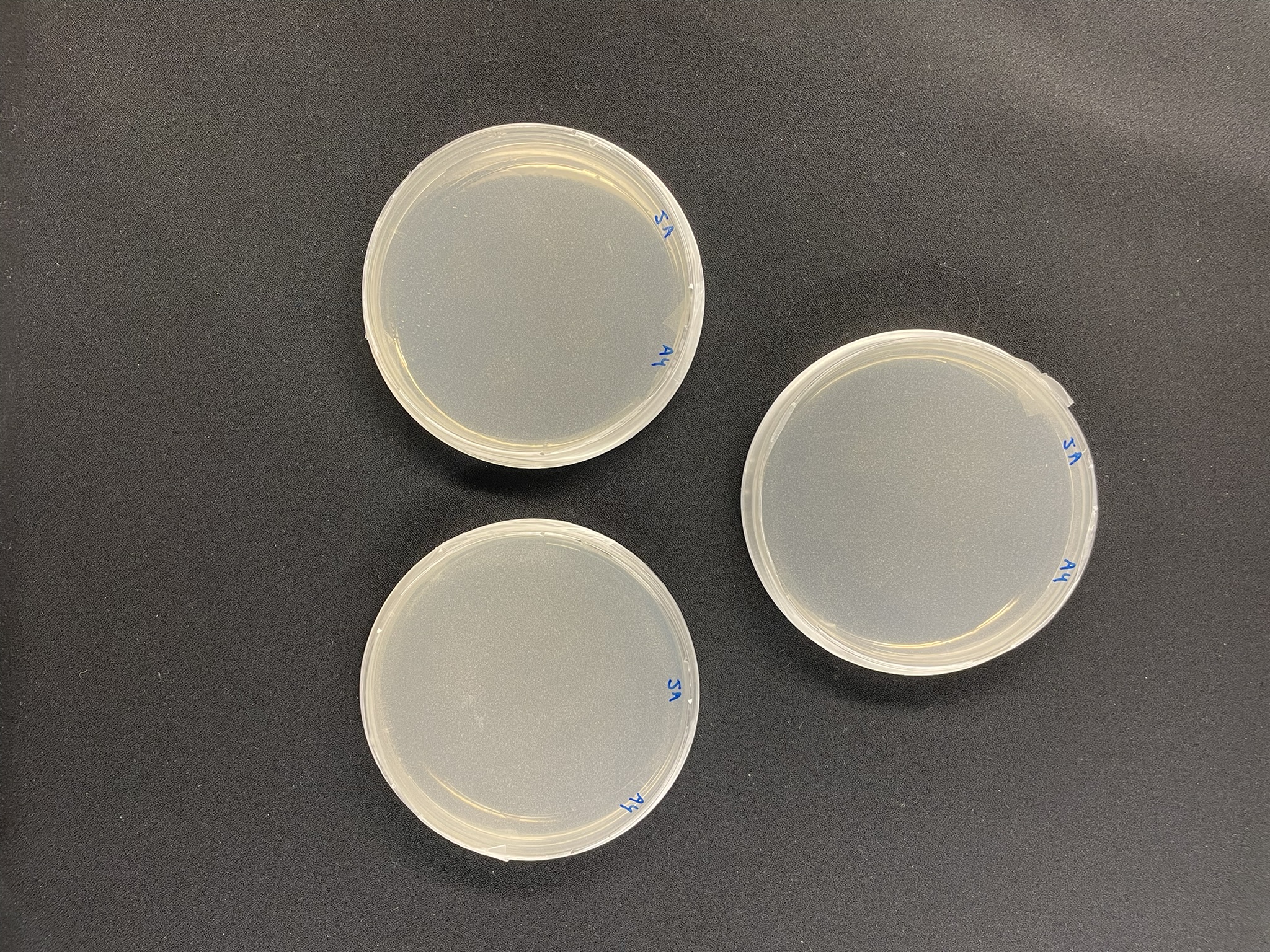

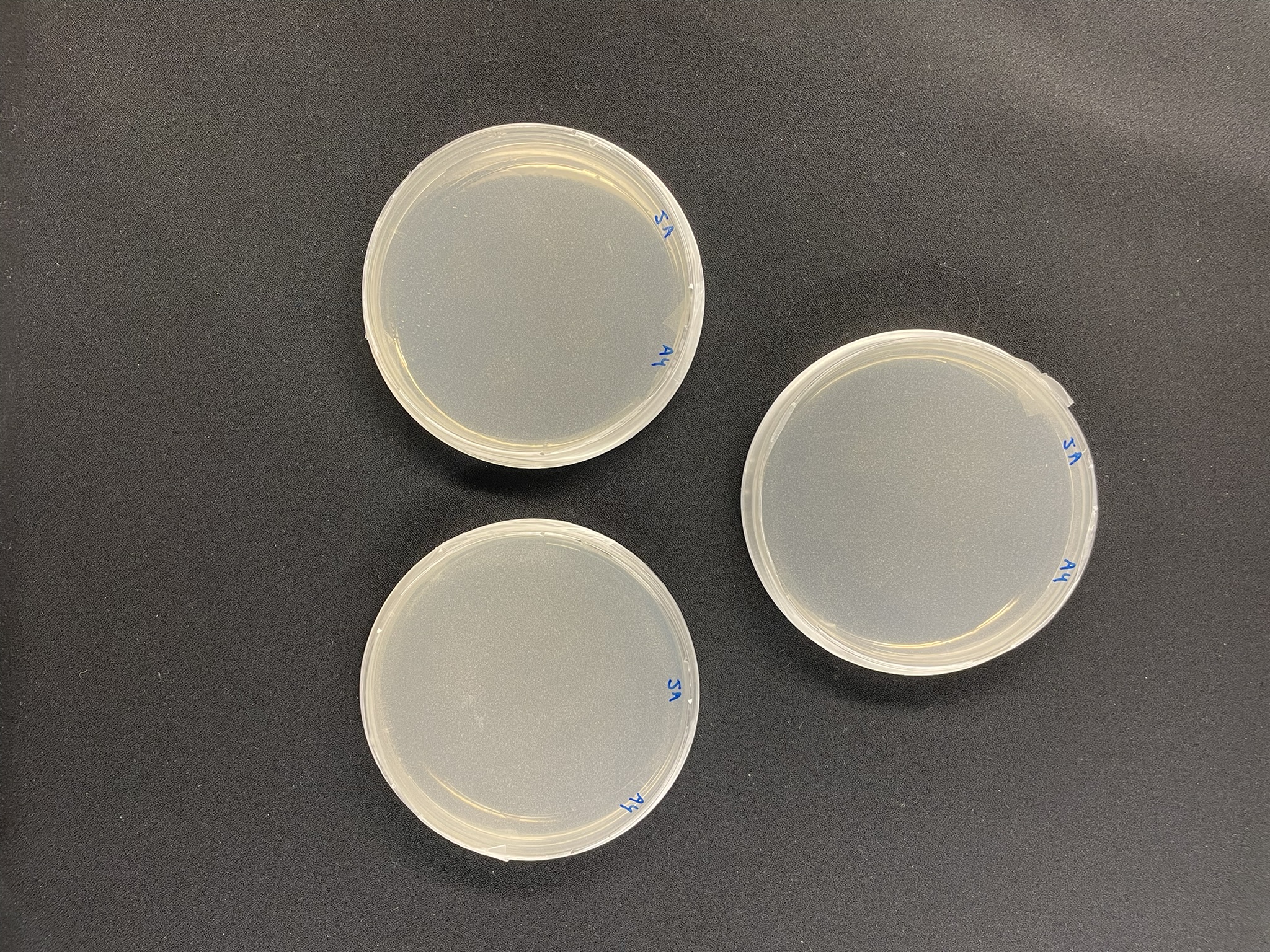

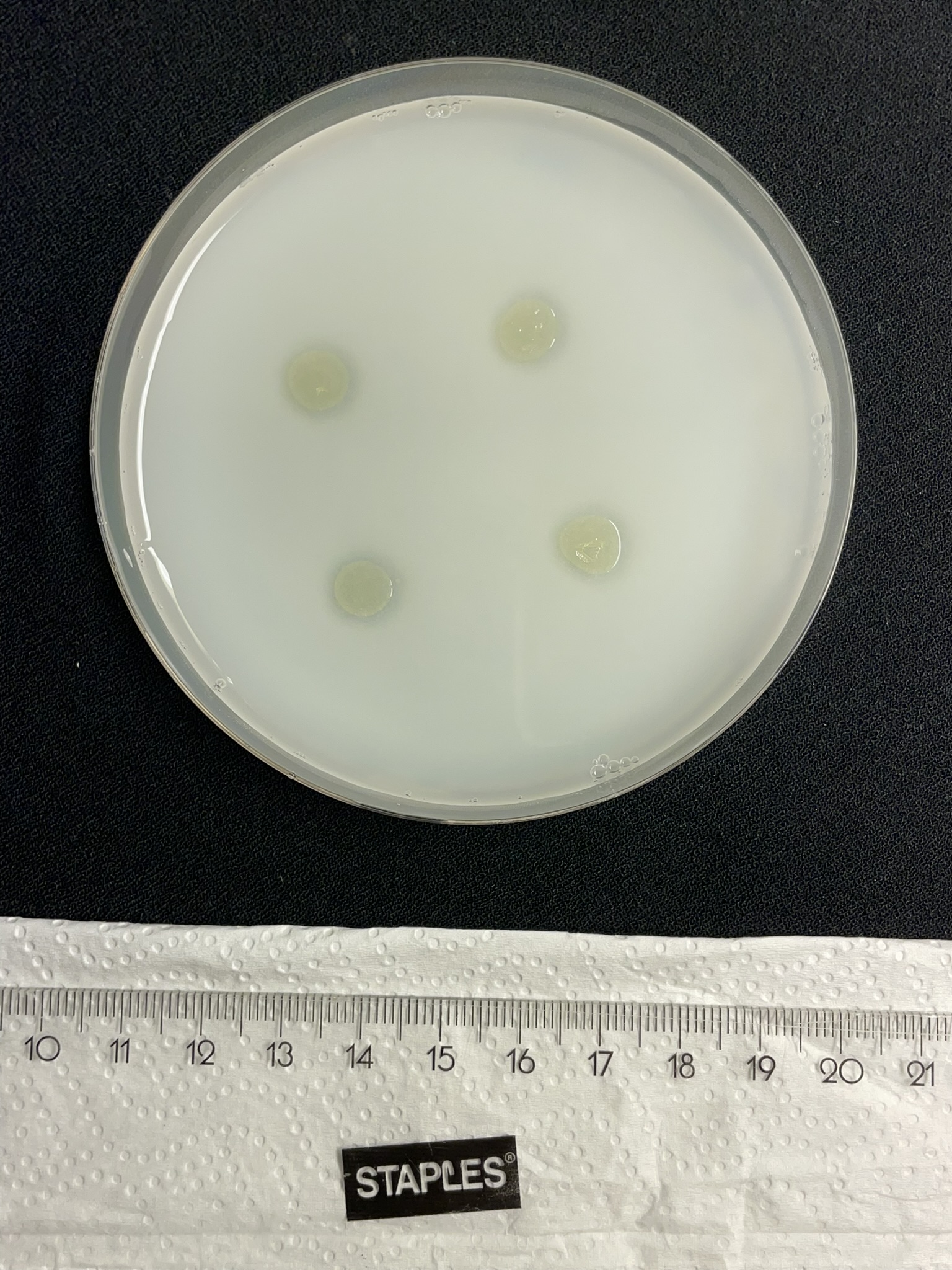

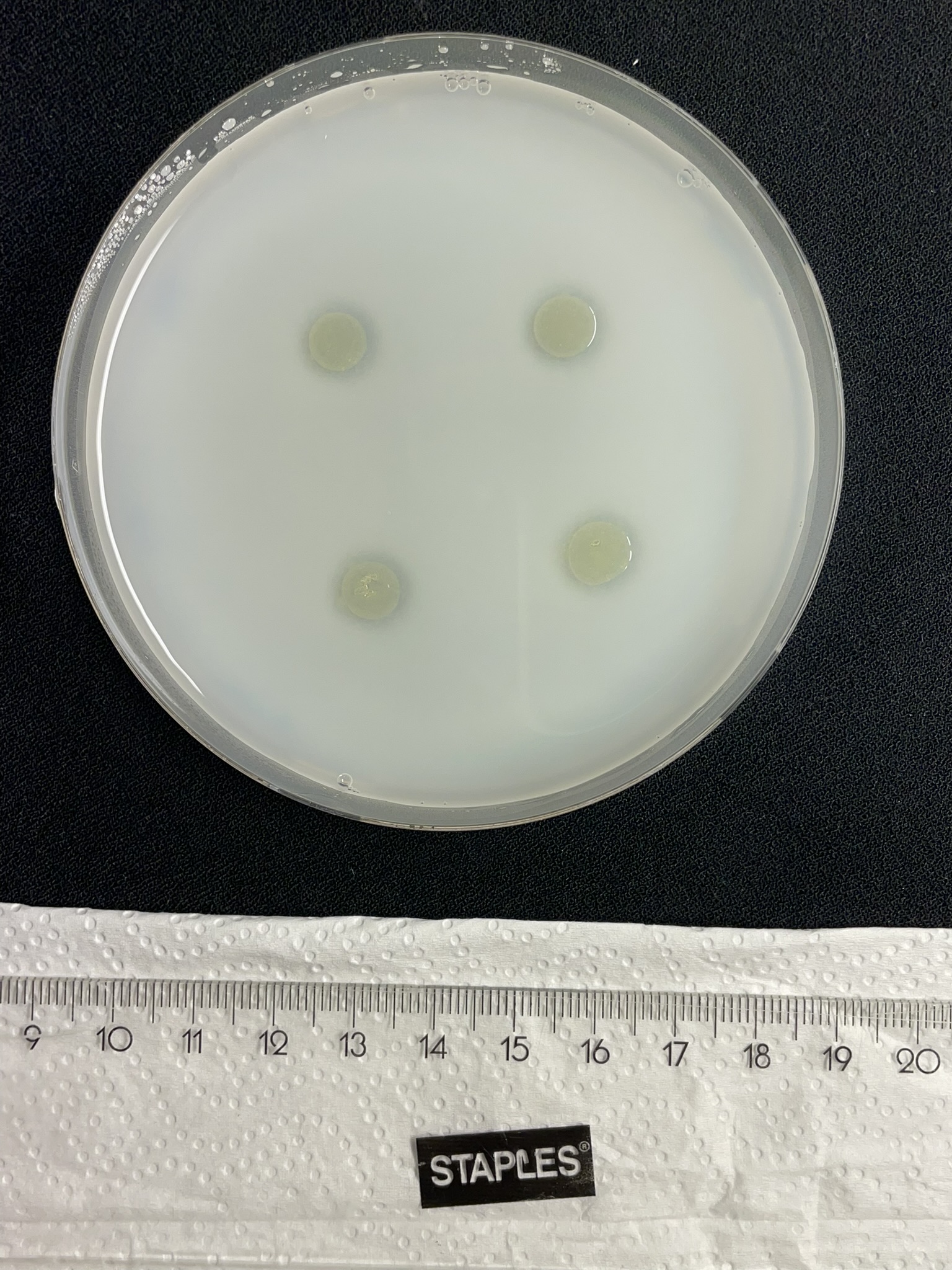

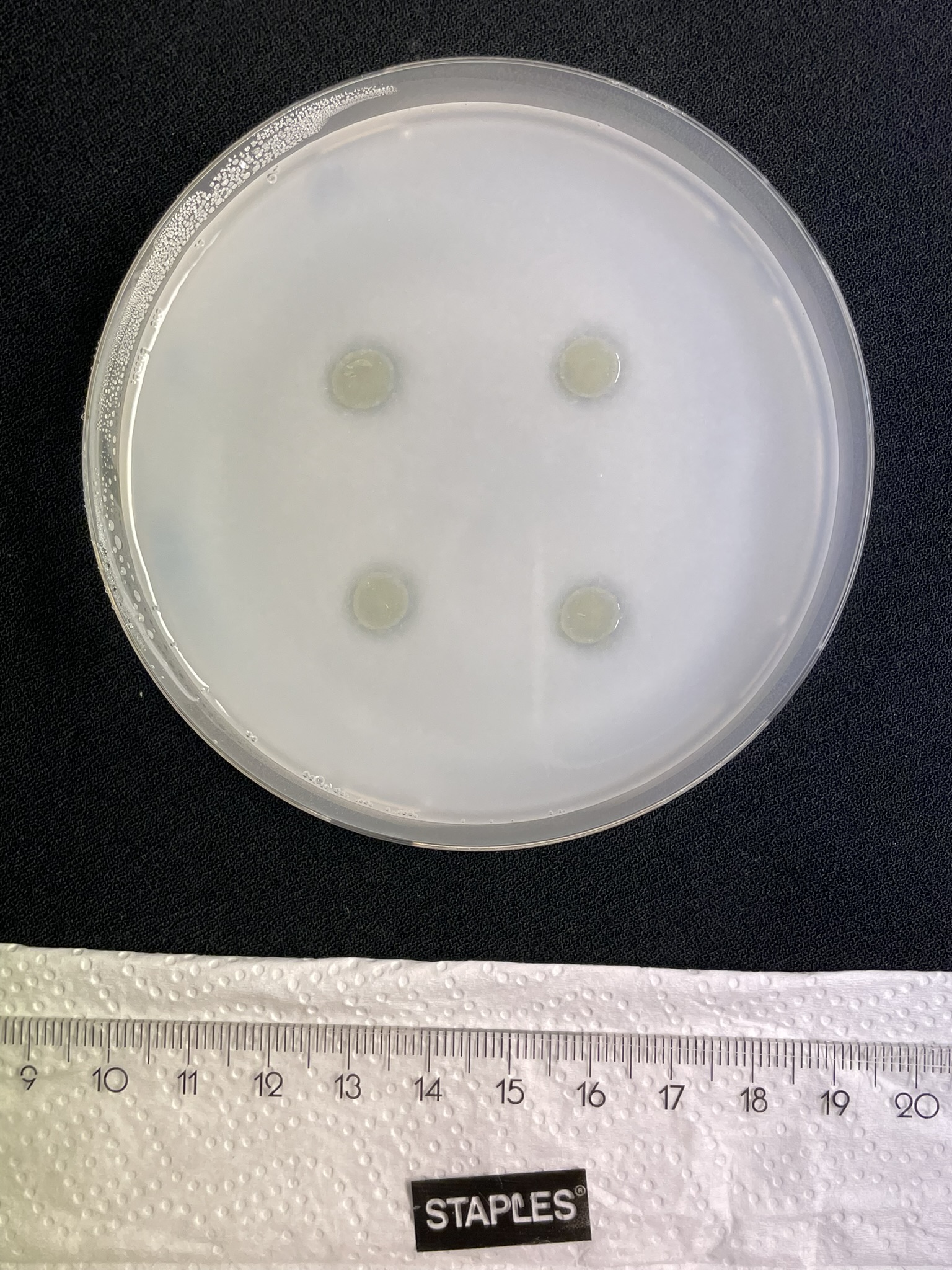


**Figure S7.** Characterization of phosphate solubilization and nitrogen fixation by *Dyella* sp. A4. (A, B) Exemplary images of Pikovskaya’s agar (A) and Jensen’s agar (B) plates inoculated with *Dyella* sp. A4.
